# Exercise type and settings, quality of life, and mental health in coronary artery disease: a network meta-analysis

**DOI:** 10.1093/eurheartj/ehae870

**Published:** 2025-01-15

**Authors:** Angel Toval, Esmée A Bakker, Joao Bruno Granada-Maia, Sergio Núñez de Arenas-Arroyo, Patricio Solis-Urra, Thijs M H Eijsvogels, Irene Esteban-Cornejo, Vicente Martínez-Vizcaíno, Francisco B Ortega

**Affiliations:** Department of Physical Education and Sports, Faculty of Sport Sciences, Sport and Health University Research Institute (iMUDS), University of Granada, Carretera de Alfacar, S/N 18071, Granada, Spain; Department of Physical Education and Sports, Faculty of Sport Sciences, Sport and Health University Research Institute (iMUDS), University of Granada, Carretera de Alfacar, S/N 18071, Granada, Spain; Department of Primary and Community Care, Radboud university medical center, P.O.Box 9101, 6500 HB Nijmegen, The Netherlands; Department of Physical Education and Sports, Faculty of Sport Sciences, Sport and Health University Research Institute (iMUDS), University of Granada, Carretera de Alfacar, S/N 18071, Granada, Spain; Health and Social Research Center, Universidad de Castilla-La Mancha, Cuenca, Spain; Department of Physical Education and Sports, Faculty of Sport Sciences, Sport and Health University Research Institute (iMUDS), University of Granada, Carretera de Alfacar, S/N 18071, Granada, Spain; AdventHealth Research Institute, Neuroscience Institute, Orlando, FL, USA; Faculty of Education and Social Sciences, Universidad Andres Bello, Viña del Mar 2531015, Chile; Department of Medical BioSciences, Exercise Physiology Research Group, Radboud university medical center, Nijmegen, The Netherlands; Department of Physical Education and Sports, Faculty of Sport Sciences, Sport and Health University Research Institute (iMUDS), University of Granada, Carretera de Alfacar, S/N 18071, Granada, Spain; CIBER de Fisiopatología de la Obesidad y Nutrición (CIBEROBN), Instituto de Salud Carlos III, Av. de Monforte de Lemos, 5, 28029 Madrid, Spain; Instituto de Investigación Biosanitaria ibs, Granada, Spain; Health and Social Research Center, Universidad de Castilla-La Mancha, Cuenca, Spain; Universidad Autónoma de Chile, Facultad de Ciencias de la Salud, Talca, Chile; Department of Physical Education and Sports, Faculty of Sport Sciences, Sport and Health University Research Institute (iMUDS), University of Granada, Carretera de Alfacar, S/N 18071, Granada, Spain; CIBER de Fisiopatología de la Obesidad y Nutrición (CIBEROBN), Instituto de Salud Carlos III, Av. de Monforte de Lemos, 5, 28029 Madrid, Spain; Faculty of Sport and Health Sciences, University of Jyväskylä, PO Box 35, FI-40014 University of Jyväskylä, Jyväskylä, Finland

**Keywords:** Exercise, Mental health, Cognition, Brain, Cardiovascular disease, Ischaemic disease, Coronary heart disease

## Abstract

**Background and Aims:**

Individuals with coronary artery disease have poorer mental health, health-related quality of life (HR-QoL), and cognition compared with (age-matched) controls. Exercise training may attenuate these effects. The aim is to systematically review and meta-analyse the effects of different exercise types and settings on brain structure/function, cognition, HR-QoL, mental health (e.g. depression, anxiety), and sleep in patients with coronary artery disease.

**Methods:**

A systematic search was conducted and a network meta-analysis compared (i) exercise types, high-intensity interval training (HIIT), HIIT + resistance (HIIT + R), moderate-intensity training (MIT), MIT + R and stretching-toning-balance training, and (ii) exercise settings, in-person and home-based.

**Results:**

A total of 42 randomized controlled trials with a parallel group design were identified, of which 36 were included in the meta-analysis. Few studies included cognition (*n* = 2), sleep (*n* = 2), and none brain structure/function (*n* = 0). Most studies examined HR-QoL (*n* = 30), depression (*n* = 15), and anxiety (*n* = 9), in which outcomes were meta-analysed. HIIT + R, HIIT, and MIT were associated with improved HR-QoL vs. no exercise (i.e. usual care) [standardized mean difference, SMD: 1.53 (95% confidence interval 0.83; 2.24), 0.44 (0.15; 0.73), and 0.44 (0.20; 0.67), respectively]. In-person exercise was associated with larger and significant improvements [HR-QoL SMD: 0.51 (0.28; 0.74), depressive SMD: −0.55 (−1.03; −0.07), and anxiety symptoms SMD: −1.16 (−2.05; −0.26)] compared with no exercise, whereas home-based programmes were not significantly associated with improvements in these outcomes. Findings were robust in secondary (i.e. intervention duration and volume) and sensitivity analyses excluding high risk of bias studies.

**Conclusions:**

Exercise training, especially in-person sessions, was associated with improved HR-QoL, depression and anxiety, independently of exercise type. However, this study raises concern about the effectiveness of home-based programmes in improving these outcomes.

Study protocol was registered in PROSPERO (ID: CRD42023402569).

## Structured Graphical Abstract

**Structured Graphical Abstract:**
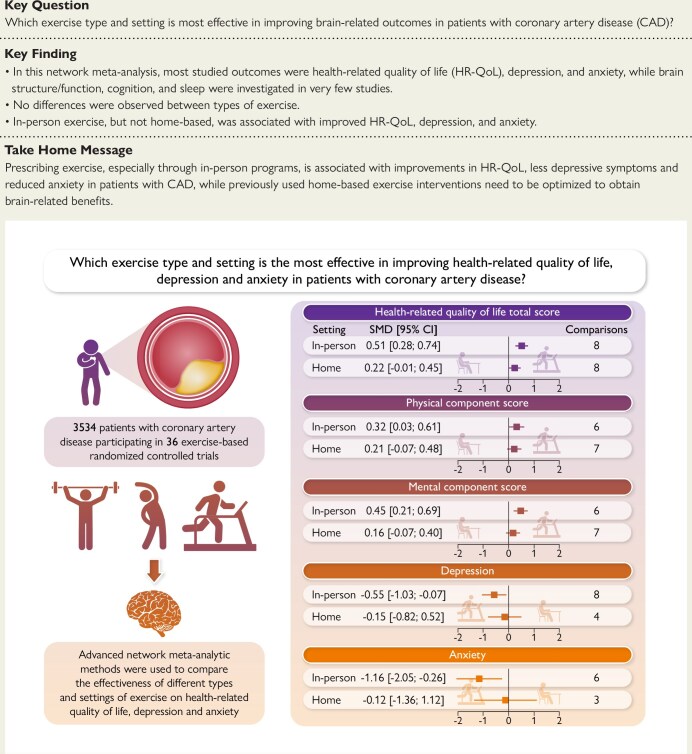
Summary of methods and key findings from the meta-analysis. The right-hand graphs display results from top to bottom for health-related quality of life: total score, physical component, and mental component, followed by depression and anxiety estimates across different exercise training settings compared with no exercise, based on network meta-analysis. The comparisons are the number of studies included in the analysis. For quality of life, positive differences indicate an improvement, whereas for depression and anxiety, negative differences indicate a reduction in symptoms. CI, confidence interval; SMD, standardized mean difference.

**See the editorial comment for this article ‘Exercise-based cardiac rehabilitation: the importance of home-based approaches', by R.S. Taylor and A. Blakemore, https://doi.org/10.1093/eurheartj/ehaf202.**

## Introduction

Coronary artery disease (CAD) is the most prevalent type of cardiovascular disease (CVD) and one of the leading causes of mortality, morbidity, and economic burden worldwide.^1–4^ Recently, several studies have revealed that the incidence of cognitive impairment in patients with CAD is markedly higher compared with age-matched healthy controls.^5–9^ Likewise, the prevalence of depression and anxiety in cardiac patients is four times higher (15%–20%) than in the general population (5%), resulting not only in an increased morbidity and mortality but also in a decreased health-related quality of life (HR-QoL).^10–14^

Brain health is a broad concept referring to the optimal functioning of behavioural and biological brain measures and the subjective experiences arising from brain function. This means that brain health includes outcomes related to neurobiological markers (e.g. structural brain morphology, brain function) and its behavioural manifestations, such as cognitive function, HR-QoL, mental health disorders (e.g. depression, anxiety), and sleep.^15,16^ Thus, it is necessary to identify effective and sustainable initiatives, which can attenuate the accelerated brain health deterioration in the CAD patient population.^5–14^

Mounting evidence supports that exercise training has important systemic and multi-organ health benefits in patients with CAD^15,17–23^ and is recommended in clinical guidelines of the American Heart Association^24^ and the European Society of Cardiology.^25^ Importantly, literature showed that exercise might also mitigate cognitive and mental health impairments observed in this population. However, the effect of different types and settings of exercise on distinct domains of brain health remains unclear. The ambiguity is partly due to the diversity (e.g. type, duration) and settings (e.g. in-person, home-based) of exercise interventions, heterogeneity in the instruments to assess different brain-related outcomes, and the predominant use of multi-component interventions.^19,26–28^ Advances in meta-analytic methods, such as network meta-analysis techniques, allow for better synthesis of existing evidence, including direct and indirect comparisons among different types and settings of exercise.

Therefore, the present systematic review and meta-analysis examines the impact of different exercise types and settings on a broad set of brain-related outcomes. More specifically, the study aims to (i) systematically identify and synthesize current literature examining the effects of physical exercise on brain-related outcomes in individuals with CAD and (ii) quantify and compare the effect of different types and settings of exercise on the most studied outcomes in CAD patients using a network meta-analysis.

## Methods

### Protocol and registration

This systematic review and network meta-analysis has been designed and reported in accordance with the Preferred Reporting Items for Systematic Reviews and Meta-Analyses (PRISMA 2020 statement),^29^ the extended PRISMA statement for Network Meta-Analyses,^30^ the Cochrane Handbook for Systematic Reviews of Interventions,^31^ and the National Institute for Health and Care Excellence (2021) guidelines.^32^ The protocol was previously registered in the international database of systematic reviews PROSPERO (ID: CRD42023402569, in March 2023).

### Search strategy and information sources

Selection of eligible studies and search strategy was structured following the PICOS (Population/Patient, Intervention, Comparison/Control, Outcome and Study design) framework strategy.^31^ Detailed description of the search strategy can be found in Supplementary data online, *File S1*. The systematic search has been conducted in different electronic databases, from inception to 12 June 2023, including Web of Science, MEDLINE (via PubMed), Scopus, EMBASE, Central Register of Controlled Trials, SPORTDiscus, and PsycINFO. In addition, we also included the Google Scholar search engine. Due to the limitation of characters allowed in Google Scholar, the search strategy was adapted, and the first 200 results sorted by relevance were extracted as previously recommended.^33^ The search strategy was developed among several researchers (A.T., E.A.B., J.B.G.-M., F.B.O., P.S.-U., and I.E.-C.) and optimized for different search engines using the Polyglot Search Translator tool from SR-Accelerator website (https://sr-accelerator.com/).^34^

### Eligibility criteria

Studies were included if they (i) assessed effects of physical activity or exercise programmes on brain structure/function, cognition, HR-QoL, mental health, and sleep in patients with CAD aged 18 years or older, (ii) were intervention studies involving any type of physical exercise, and (iii) included at least two conditions/intervention groups (i.e. exercise vs. control or exercise vs. exercise), since we were interested in comparisons. Studies were excluded when (i) they had a quasi-experimental design without comparison group/condition, (ii) they were animal studies, (iii) they included patients with other CVDs, unless the study population contained >85% of patients with CAD, (iv) they consisted of grey literature (e.g. letters to the editor, congress abstracts, unpublished studies), (v) they were not published in English, Spanish, Dutch, or Portuguese, and (vi) study arms were excluded when they combined exercise with other therapies (e.g. pharmacologic treatment, diet, psychological, or educational therapy) unless the intervention groups only differed on the exercise component.

### Study selection process

**Figure 1:**
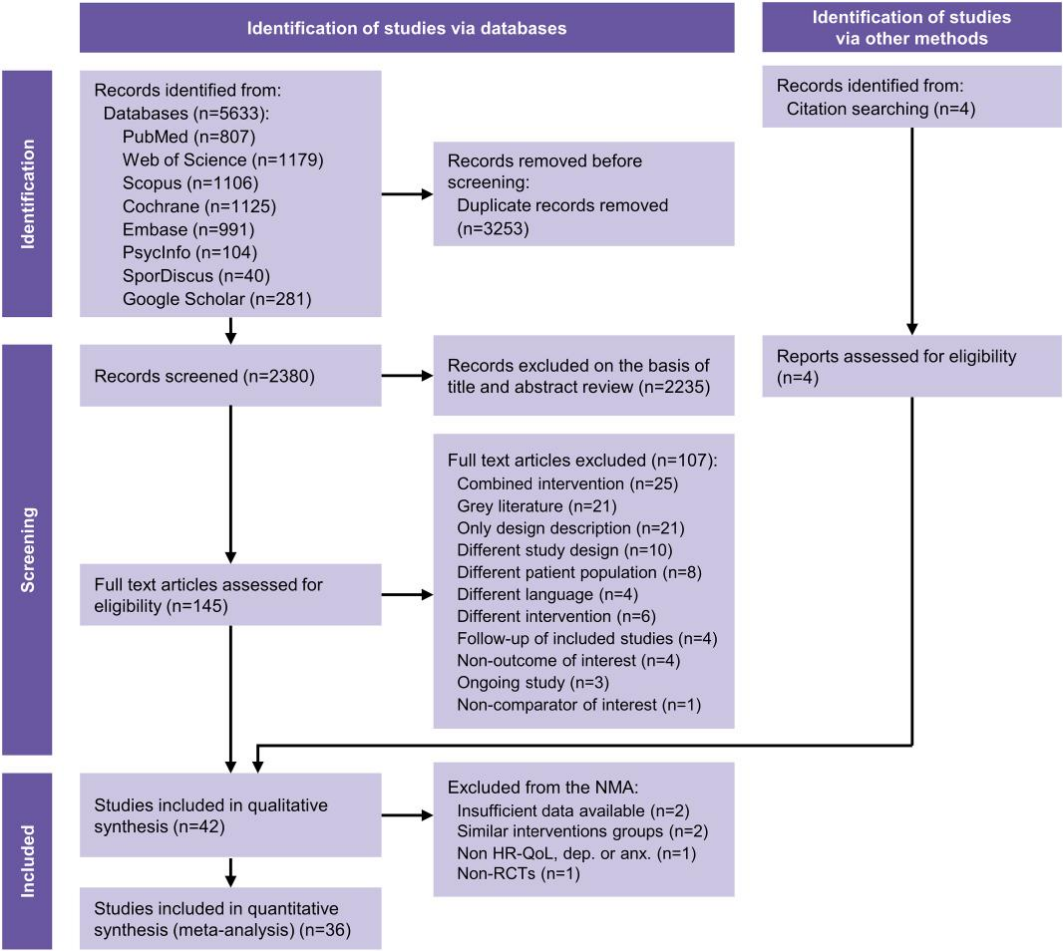
Preferred Reporting Items for Systematic Reviews and Meta-Analyses study selection and flow chart. NMA, network meta-analysis; HR-QoL, health-related quality of life; dep., depression; anx., anxiety; RCT, randomized controlled trial.

The study identification, screening, and inclusion of eligible studies were carried out by three reviewers (A.T., E.A.B., and J.B.G.-M.) independently and followed the PRISMA flow chart (*Figure 1*). After a comprehensive search of multiple databases, duplicate publications were removed. Next, the titles and abstracts were screened, and then the full texts were screened against inclusion/exclusion criteria. When necessary, an additional reviewer (F.B.O.) resolved discrepancies between reviewers prior to reaching a consensus. Covidence software was used to manage records throughout the study selection/screening process.^35^ For the quantitative synthesis (network meta-analysis), we only included the studies assessing HR-QoL, depression, and/or anxiety due to the low number of studies assessing other brain-related outcomes.

### Data extraction and processing

Data from included records were extracted by two researchers independently (A.T. and E.A.B.) using a standardized, pre-specified, and piloted form. Based on the PICOS strategy, we extracted information on the population and study characteristics, intervention, comparison, and outcomes. More details are provided in Supplementary data online, *File S2* (extended methods).

### Categories of different types of exercise

Many studies have shown the differential physiological adaptations derived from different types of exercise, such as aerobic training, resistance training, and the combination of aerobic and resistance training.^36,37^ Furthermore, studies suggested differential effects on physiological outcomes as response to high-intensity interval training (HIIT) and moderate-intensity training (MIT)^38,39^ justifying that HIIT and MIT could be considered as two different types of exercise.^40^ We categorized the studies into five exercise types based on available literature and the designs of the network plots: HIIT, MIT, HIIT with resistance training (HIIT + R), MIT with resistance training (MIT + R), and stretching-toning-balance training (STBT; *Table 1*). The following information on the exercise interventions was extracted from each study: exercise type, setting (i.e. in-person or home-based), duration of the intervention, frequency (sessions/week), duration of the sessions, and exercise intensity (i.e. moderate or high). Due to the large heterogeneity of reporting exercise intensity (e.g. % of maximum heart rate, % of maximum oxygen uptake, rate of perceived exertion) and the number of repetitions during resistance exercise, we decided not to extract or analyse this information.

**Table 1:** Characteristics of the exercise types included in the network meta-analysis

MIT	Aerobic moderate continuous training refers to exercise interventions that increase heart rate and energy expenditure with a constant but moderate intensity. It includes activities such as walking, jogging and cycling, outdoor or indoor
HIIT	Aerobic high-intensity interval training refers to exercise interventions that consist of short and repeated bouts performed at high intensity (i.e. close to maximal intensities), interspersed by periods of lower intensity exercise or rest for recovery
MIT + R	Aerobic moderate continuous training combined with resistance training. Resistance exercise refers to exercise interventions that aim to increase muscular strength and power, including exercises performed with dumbbells, Thera-bands, or pneumatic machines
HIIT + R	Aerobic high-intensity interval training combined with resistance training. Resistance exercise refers to exercise interventions that aim to increase muscular strength and power, including exercises performed with dumbbells, Thera-bands, or pneumatic machines
STBT	Stretching-toning-balance training. Refers to physical exercises that include Tai-Chi, yoga, qigong, among others, excluding interventions focused on or combined with mind exercises

### Data processing

Change-from-baseline values (to post-intervention) were used as main outcome. We calculated missing standard deviations (SDs) based on the standard error, 95% confidence intervals (CIs), or inter-quartile range.^31^ As most studies did not report change-from-baseline values, we estimated the change-from-baseline with corresponding SDs using the pre- and post-intervention values with an imputed correlation of 0.66.^31^ This imputed correlation was based on the weighted average correlation from some of the included studies reporting change-from-baseline values for the study outcomes (*n* = 7). For the sensitivity analyses, we used a more conservative correlation of 0.50.^32^ When the change-from-baseline and the pre- and post-intervention values were not reported, we contacted the study authors to request this information (*n* = 4).^41–44^ For studies including only a visual presentation (*n* = 1),^45^ we used a graphical software programme (WebPlotDigitizer, version 4.6, https://automeris.io/WebPlotDigitizer)^46,47^ and averaged the results of both reviewers (A.T. and E.A.B.). More detailed information about the data processing and calculating the overall and mental component score (MCS) and physical component score (PCS) of HR-QoL are described in the extended methods (see Supplementary data online, *File S2*).

### Risk of bias

Risk of bias was assessed for randomized controlled trials (RCTs) using the Cochrane risk-of-bias tool (RoB 2) for randomized trials.^48^ Two reviewers (A.T. and E.A.B.) individually assessed the risk of bias, and disagreements were solved between the two reviewers or by consulting the third reviewer (F.B.O.) if necessary. To evaluate the certainty of the evidence, we used the Confidence in Network Meta-Analysis.^49,50^

### Statistical analyses

Our network meta-analysis compared the effect of different (i) exercise types, i.e. HIIT, HIIT + R, MIT, MIT + R, and STBT, and (ii) exercise settings i.e. in-person and home-based, on the most studied outcomes (i.e. HR-QoL, depression, and anxiety). Of the 37 studies focused on these outcomes, all but one were RCTs. Therefore, we excluded the single non-RCT^51^ from the analyses to obtain more robust and homogeneous estimates based only on the best study design, i.e. RCTs. We created network geometry graphs to examine the shape of the network of HR-QoL, PCS, MCS, depression, and anxiety. We excluded outcomes from the network meta-analysis when a star-shaped network occurred (i.e. one intervention is compared with all others but direct comparisons between these others are absent). Next, we conducted a random-effects network meta-analysis of which specific details are provided in the extended methods (see Supplementary data online, *File S2*). The results were presented using league tables and forest plots of the network estimates using no exercise as reference category. The main network meta-analysis assumptions (i.e. transitivity and consistency) were checked (see Supplementary data online, *File S2*). We assessed statistical heterogeneity in each pairwise and network meta-analysis comparison using *t*² and *I*² statistics. The *I*^2^ statistic was interpreted as not important (0%–30%), moderate (30%–50%), substantial (50%–75%), or considerable (75%–100%), and the corresponding *P*-values were also considered. Ranking probabilities for different exercise interventions were estimated using *P*-scores. *P*-scores were measured on a scale from 0 (worst) to 1 (best). Finally, we created funnel plots to explore potential presence of publication bias. We did not perform statistical testing for publication bias, because there were <10 studies available per direct comparison.^52^

Based on the results of the main analysis (i.e. no clear differences between exercise intensity such as HIIT vs. MIT), we examined the effect of in-person vs. home-based exercise vs. no exercise by creating a new network. Since the difference between HIIT and MIT was absent but the difference between exercise settings (i.e. in-person vs. home-based) was clearly present, we decide to create the following groups for the additional analyses on duration and total volume: in-person exercise >12 weeks, in-person exercise ≤12 weeks, home-based exercise >12 weeks, and home-based exercise ≤12 weeks and in-person exercise >24 h (total volume is hours trained during the intervention), in-person exercise ≤24 h, home-based exercise >24 h, and home-based exercise ≤24 h. Study characteristics of *Table 2* were used to create cut-off values for duration (i.e. 12 weeks) and total volume (i.e. 24 h). Sensitivity analyses were performed using a conservative correlation (0.5) and excluding studies with a high risk of bias. The analyses were performed with R (version 4.2.3) using the packages ‘netleague’ and ‘netmeta’. Statistical significance (two sided) was set at *P* < .05.

**Table 2:** Characteristics of the studies included in the network meta-analysis

Study	Sample size (% women)	Age, years	Intervention type	Duration of exercise interventions	Time after event^a^	Instruments		
						HR-QoL	Depression	Anxiety
Avila *et al*.^53^	84 (11%)	61 ± 8	(i) Home MIT, (ii) in-person MIT + R, (iii) nEX	12 weeks	3	36-Item Short Form Survey (SF-36)		
Belardinelli *et al*.^54^	118 (16%)	57 ± 10	(i) In-person MIT, (ii) nEX	26 weeks	4	Duke Activity Status Index (DASI), Medical Outcomes Study (MOS)		
Blumenthal *et al*.^55^	134 (31%)	63 ± 10	(i) In-person MIT, (ii) SM^b^, (iii) nEX	16 weeks	2, 3		Beck's Depression Inventory II (BDI-II)	The State-Trait Anxiety Inventory (STAI-State)
Blumenthal, *et al*.^56^	101 (32%)	64 ± NR	(i) In-person MIT, (ii) MED^b^, (iii) nEX	16 weeks	4		Hamilton Depression Rating Scale (HAM-D)	
Blumenthal *et al*.^57^	128 (29%)	65 ± 10	(i) In-person MIT, (ii) MED^b^, (iii) nEX	12 weeks	4		BDI-II, Hospital Anxiety and Depression Scale (HADS-D)	HADS-A, STAI-State
Brouwers *et al*.^42^	300 (11%)	61 ± 10	(i) Home MIT + R, (ii) in-person MIT + R	13 weeks	2	European Quality of Life 5 Dimensions 5 Level Version (EQ-5D-5L), European Quality of Life Visual Analogue Scale (EQ-VAS)		
Conraads *et al*.^58^	174 (20%)	58 ± 9	(i) In-person HIIT, (ii) in-person MIT	12 weeks	2	12-Item Short Form Survey (SF-12)		
Currie *et al*.^59^	19 (7%)	65 ± 8	(i) In-person MIT, (ii) in-person HIIT	12 weeks	2	SF-36		
Deka *et al*.^60^	90 (24%)	69 ± 5	(i) In-person HIIT + R, (ii) nEX	8 weeks	4	SF-36		
Deng *et al*.^61^	70 (14%)	81 ± 4	(i) In-person MIT, (ii) nEX	12 weeks	1	SF-12	Zung Self-Rating Depression Scale (SDS)	Self-Rating Anxiety Scale (SAS)
Dor-Haim *et al*.^62^	29 (0%)	R 47–69	(i) In-person MIT, (ii) in-person MIT + R	12 weeks	2	SF-12		
Fang *et al*.^63^	67 (37%)	61 ± 10	(i) Home MIT, (ii) nEX	6 weeks	2	SF-36	Cardiac Depression Scale (CDS)	
Ghisi *et al*.^64^	115 (29%)	60 ± 9	(i) In-person MIT + R + Edu^b^, (ii) in-person MIT + R, (iii) nEX	26 weeks	2		Patient Health Questionnaire (PHQ-9)	
Giggins *et al*.^65^	17 (18%)	70 ± 8	(i) Home MIT + R, (ii) in-person MIT + R	8 weeks	2	SF-12		
Jaureguizar *et al*.^66^	72 (15%)	58 ± 11	(i) In-person HIIT, (ii) in-person MIT	8 weeks	2	SF-36, MacNew Heart Disease health-related quality of life (MacNew)		
Jiang *et al*.^67^	120 (60%)	62 ± 10	(i) In-person STBT, (ii) nEX	13 weeks	1	SF-36	HAM-D, SDS	HAM-A, SAS
Johnson *et al*.^68^	154 (100%)	(i) 64 (IQR 14) (ii) 62 (IQR 12)	(i) Home MIT, (ii) nEX	12 weeks	3	MacNew		
Kristiansen *et al*.^69^	142 (17%)	67 ± 9	(i) In-person HIIT, (ii) nEX	12 weeks	3	SF-36		
Lee *et al*.^70^	31 (100%)	68 ± 9	(i) Home HIIT, (ii) home MIT	24 weeks	2		Center for Epidemiologic Studies Depression Scale (CES-D)	
Maddison *et al*.^43^	171 (19%)	60 ± 9	(i) Home MIT, (ii) nEX	24 weeks	2, 3	SF-36, EQ-5D-5L		
Maddisson *et al*.^71^	140 (14%)	61 ± 13	(i) Home MIT, (ii) in-person MIT	12 weeks	2	EQ-5D		
Madssen *et al*.^72^	49 (27%)	(i) 64 (R 47–78) (ii) 59 (R 42–71)	(i) Home HIIT, (ii) nEX	52 weeks	3	MacNew		
McGregor *et al*.^73^	382 (7%)	59 ± 10	(i) In-person HIIT, (ii) in-person MIT	8 weeks	2	EQ-5D-5L, EQ-VAS		
Moholdt *et al*.^74^	26 (20%)	63 ± 8	(i) In-person MIT + R, (ii) home HIIT	(i) 4 weeks, (ii) 26 weeks	2	MacNew		
Okur *et al*.^75^	20 (10%)	61 ± 5	(i) In-person MIT, (ii) in-person HIIT, (iii) In-person HIIT	5 weeks	2	SF-36, MacNew		
Reed *et al*.^76^	130 (15%)	61 ± 7	(i) In-person HIIT, (ii) in-person MIT, (iii) In-person MIT	12 weeks	2	SF-36, HeartHR-QoL	BDI-II	
Salmoirago-Blotcher *et al*.^77^	29 (27%)	68 ± 10	(i) In-person STBT, (ii) In-person STBT	(i) 12 weeks, (ii) 24 weeks	2	SF-8		
Schönfelder *et al*.^78^	60 (26%)	61 ± 12	(i) In-person MIT, (ii) in-person HIIT, (iii) in-person MIT	6 weeks	2	MacNew	HADS-D	HADS-A
Snoek *et al*.^79^	118 (18%)	60 ± 10	(i) Home MIT, (ii) nEX	26 weeks	2	Dutch version of the Quality of Life after Myocardial Infarction	HADS-D	HADS-A
Takroni *et al*.^80^	79 (19%)	55 ± 7	(i) In-person MIT, (ii) home MIT, (iii) nEX	8 weeks	3	SF-36	HADS-D	HADS-A
Taylor *et al*.^81^	93 (16%)	65 ± 8	(i) In-person HIIT, (ii) in-person MIT	52 weeks	2	MacNew		
Terada *et al*.^82^	130 (15%)	61 ± 7	(i) In-person HIIT, (ii) in-person MIT, (iii) In-person MIT	12 weeks	2	SF-36, HeartHR-QoL	BDI-II	
Vieira *et al*.^83^	33 (NR)	58 ± 9	(i) Home MIT + R, (ii) home MIT + R, (iii) nEX	26 weeks	3	MacNew	The Depression, Anxiety and Stress Scale - 21 Items (DASS-21)	DASS-21
Villafaina *et al*.^84^	21 (0%)	56 ± 6	(i) In-person HIIT, (ii) in-person MIT	12 weeks	3	SF-12		
Yakut *et al*.^28^	21 (14%)	59 ± 5	(i) Home HIIT, (ii) home MIT	12 weeks	2,3	MacNew		
Yu *et al*.^45^	204 (24%)	64 ± 11	(i) In-person MIT, (ii) nEX	8 weeks	2	SF-36	Symptoms Questionnaire	Symptoms Questionnaire

Data are presented as mean ± SD, mean [range (R)], or median (IQR), as appropriate.

NR, not reported; RCT, randomized controlled trial; nEX, non-exercise; MIT, moderate-intensity training; HIIT, high-intensity interval training; STBT, stretching-toning-balance training; SM, stress management programme; MED, medication treatment; ses, sessions; HR-QoL, health-related quality of life; Dep, depression; Anx, anxiety.

^a^Time after event: (i) eligible for cardiac rehabilitation and within 1 month after event, (ii) eligible for cardiac rehabilitation or within 2–6 months after event, (iii) after cardiac rehabilitation or 6 months after the event, and (iv) not reported.

^b^Intervention arm was excluded from the network meta-analysis because it included a pharmacological or psychological treatment or additionally included education besides exercise.

## Results

### Synthesis of studies found in the systematic search

Our systematic search identified 2380 studies, of which 145 underwent full-text review. Additionally, four studies were identified from manual citation searching. A total of 42 studies^28,41–45,51,53–87^ were included in the qualitative synthesis and 36 studies^28,42,43,45,53–76,78–84^ were included in the quantitative synthesis (meta-analysis) as summarized in *Figure 1*. From the 42 studies included in the qualitative synthesis, the most assessed outcome was HR-QoL (36 studies, 86%; *n* = 3476 subjects), followed by depression (17 studies, 40%; *n* = 2032) and anxiety (9 studies, 21%; *n* = 946). Two studies evaluated cognitive function, two studies examined sleep, and none of the studies evaluated neurobiological markers, such as structure and function using neuroimaging techniques or any other methods.

### Characteristics of studies included in the network meta-analysis

The 36 studies included in the meta-analysis were RCTs with parallel group design using the outcomes HR-QoL (*n* = 30), depression (*n* = 15), and anxiety (*n* = 9). Overall, 3534 participants with CAD were randomly assigned to exercise (*n* = 2707) or no exercise (*n* = 827). In all studies, the ‘no exercise’-intervention arm consisted of usual care. The mean age was 62 (SD 9.3) years, and the mean percentage of women was 23%. Most interventions were performed when patients were eligible for cardiac rehabilitation (CR) or within 2–6 months after the cardiovascular event (23 studies, 64% of the total included). The most common exercise was MIT (26 studies, 72%, *n* = 1479), followed by HIIT (15 studies, 42%, *n* = 621), MIT + R (7 studies, 19%, *n* = 473), STBT (2 studies, 6%, *n* = 89), and HIIT + R (1 study, 3%, *n* = 45). In-person exercise was performed in 28 studies (78%, *n* =2065), while home-based interventions were performed in 14 studies (39%, *n* = 642; *Table 2*).

We found no clear evidence of violations of the transitivity assumption in the studies characteristics (*Table 2*) suggesting that the included participants had similar characteristics (i.e. effect modifiers) across comparisons. In terms of risk of bias, most studies were rated with some concerns (18 studies; 50% of the included studies), followed by a low risk (10 studies, 28%) and a high risk (8 studies, 22%, see Supplementary data online, *Figures S1* and *S2*).

### Structure of network

**Figure 2:**
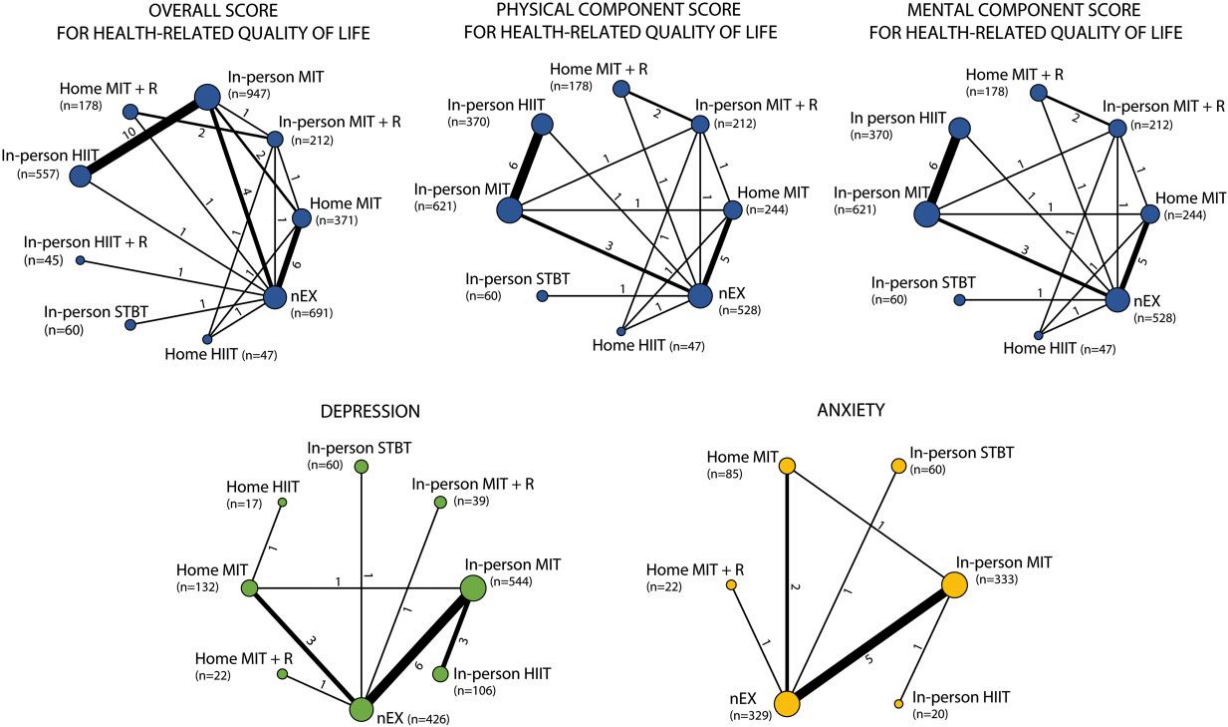
Network of eligible comparisons for different brain-related outcomes. The width of the lines is proportional to the number of trials comparing each pair of treatments. The size of the nodes is proportional to the number of participants. The outcomes were assessed as follows: (i) overall score of health-related quality of life in *n* = 30, (ii) PCS of health-related quality of life in *n* = 25, (iii) MCS of health-related quality of life in *n* = 25, (iv) depression in *n* = 15, and (v) anxiety in *n* = 9 studies. HIIT, high-intensity interval training; HIIT + R, high-intensity interval training plus resistance training; MIT, moderate-intensity training; MIT + R, moderate-intensity training plus resistance training; STBT, stretching-toning-balance training; nEX, no exercise

*Figure 2* shows the networks of eligible comparisons for HR-QoL, depression, and anxiety of the main analyses regarding exercise types. The network graphs of HR-QoL were well connected, but for depression and anxiety, we found a star network that prevented us from running analyses including indirect comparisons. Supplementary data online, *Figure S3* presents the networks for the analyses regarding exercise setting (i.e. in-person vs. home-based vs. no exercise), in which all networks were well connected. For the secondary analyses focused on intervention duration and total exercise volume, the network plots were well connected for all HR-QoL outcomes, but not for the depression and anxiety (i.e. star shaped).

### Different types of exercise and health-related quality of life

**Figure 3:**
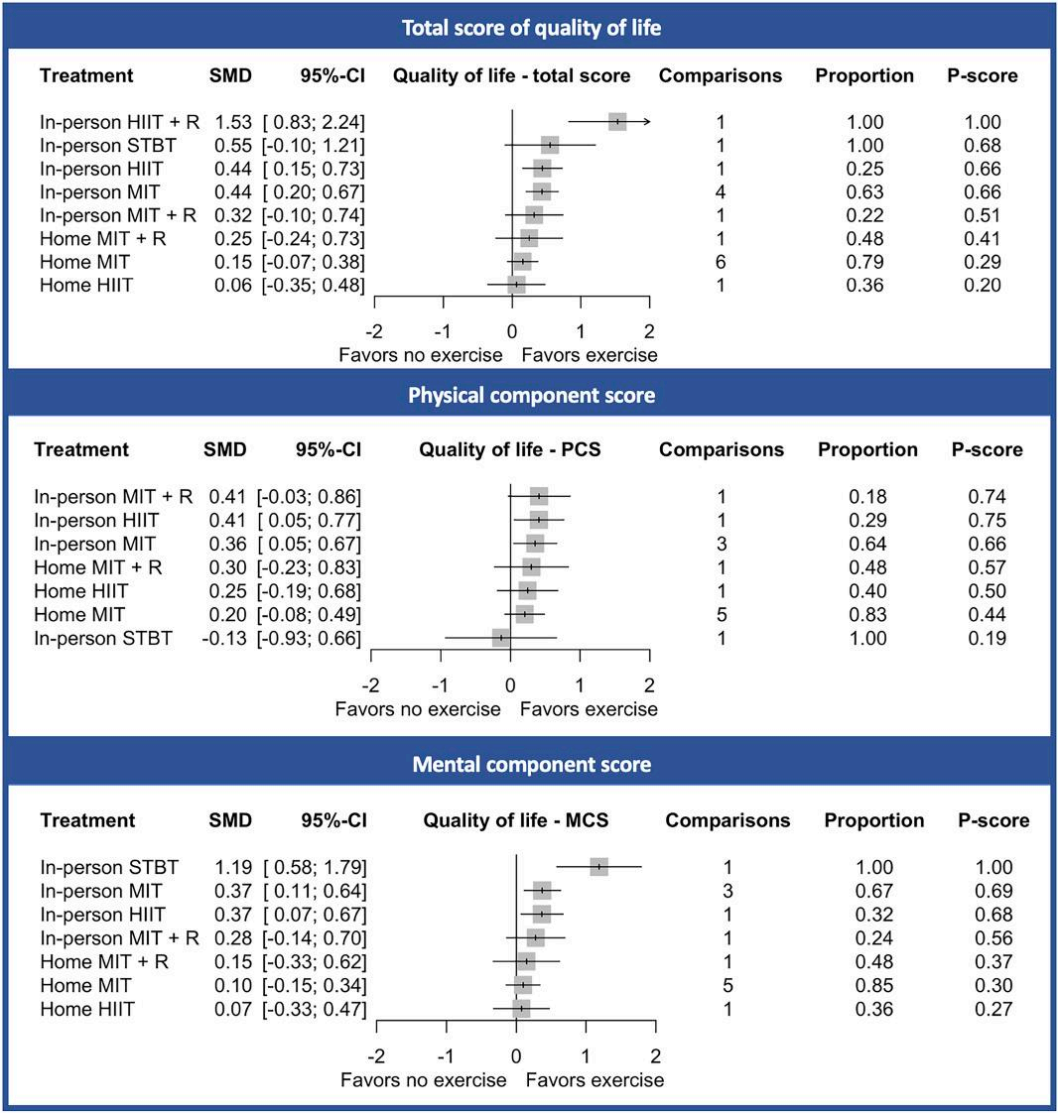
Health-related quality of life estimates for total, physical, and mental component scores for different types of exercise compared with no exercise (usual care) using the network meta-analysis. The comparisons are the number of studies included in the analyses. The proportion represents the amount of direct evidence that was used to determine the effect estimates. *P*-scores were scaled from 0 (worst) to 1 (best). SMD, standardized mean difference; 95% CI, 95% confidence interval; HIIT, high-intensity interval training; HIIT + R, high-intensity interval training plus resistance training; MIT, moderate-intensity training; MIT + R, moderate-intensity training plus resistance training; STBT, stretching-toning-balance training; PCS, physical component score; MCS, mental component score

Based on the network meta-analysis, in-person HIIT + R was associated with the largest, statistically significant improvement for the total score of HR-QoL [standardized mean difference (SMD) 1.53, 95% CI 0.83; 2.24, *Figure 3* and *Table 3*] compared with no exercise, followed by in-person HIIT (SMD 0.44, 95% CI 0.15; 0.73) and in-person MIT (SMD 0.44, 95% CI 0.20; 0.67). However, in-person HIIT + R was solely based on one study, whereas in-person HIIT and MIT were based on multiple comparisons. No differences were found in HR-QoL changes between HIIT and MIT, either in-person (SMD 0.00, 95% CI −0.20; 0.21) or home-based (SMD −0.09, 95% CI −0.50; 0.32, *Table 3*).

**Table 3:** League table with pooled standardized mean difference for different exercise types and health-related quality of life; total, physical, and mental component score

Health-related quality of life—total score								
**In-person HIIT**		0.04 (−0.18; 0.25)						0.21 (−0.36; 0.78)
**−1.09 (−1.86; −0.33)**	**In-person HIIT + R**							**1.53 (0.83; 2.24)**
0.00 (−0.20; 0.21)	**1.10 (0.35; 1.84)**	**In-person MIT**	−0.36 (−1.13; 0.40)			0.31 (−0.09; 0.71)		**0.60 (0.31; 0.90)**
0.12 (−0.35; 0.59)	**1.21 (0.39; 2.03)**	0.11 (−0.32; 0.55)	**In-person MIT + R**		−0.09 (−0.88; 0.70)	0.25 (−0.58; 1.08)	0.06 (−0.41; 0.53)	−0.03 (−0.92; 0.85)
−0.11 (−0.83; 0.61)	**0.98 (0.01; 1.95)**	−0.12 (−0.82; 0.58)	−0.23 (−1.01; 0.55)	**In-person STBT**				0.55 (−0.10; 1.21)
0.38 (−0.11; 0.87)	**1.47 (0.65; 2.29)**	0.37 (−0.08; 0.83)	0.26 (−0.24; 0.76)	0.49 (−0.29; 1.27)	**Home HIIT**	−0.11 (−0.68; 0.47)		−0.18 (−0.88; 0.51)
0.29 (−0.04; 0.62)	**1.38 (0.64; 2.12)**	**0.28 (0.01; 0.56)**	0.17 (−0.26; 0.60)	0.40 (−0.29; 1.09)	−0.09 (−0.50; 0.32)	**Home MIT**		0.16 (−0.09; 0.41)
0.19 (−0.35; 0.74)	**1.29 (0.43; 2.15)**	0.19 (−0.33; 0.70)	0.07 (−0.34; 0.48)	0.31 (−0.51; 1.12)	−0.19 (−0.77; 0.40)	−0.09 (−0.60; 0.42)	**Home MIT + R**	0.22 (−0.49; 0.92)
**0.44 (0.15; 0.73)**	**1.53 (0.83; 2.24)**	**0.44 (0.20; 0.67)**	0.32 (−0.10; 0.74)	0.55 (−0.10; 1.21)	0.06 (−0.35; 0.48)	0.15 (−0.07; 0.38)	0.25 (−0.24; 0.73)	**nEX**

Health-related quality of life—physical component score							
**In-person HIIT**	0.05 (−0.20; 0.29)						0.47 (−0.19; 1.13)
0.06 (−0.18; 0.29)	**In-person MIT**	−0.37 (−1.12; 0.38)			0.34 (−0.31; 0.98)		**0.43 (0.04; 0.82)**
0.00 (−0.51; 0.51)	−0.06 (−0.53; 0.42)	**In-person MIT + R**	.	0.07 (−0.68; 0.82)	0.37 (−0.55; 1.30)	0.03 (−0.51; 0.57)	−0.03 (−1.08; 1.01)
0.54 (−0.33; 1.42)	0.49 (−0.37; 1.35)	0.55 (−0.37; 1.46)	**In-person STBT**				−0.13 (−0.93; 0.66)
0.17 (−0.37; 0.70)	0.11 (−0.39; 0.61)	0.17 (−0.34; 0.67)	−0.38 (−1.29; 0.53)	**Home HIIT**	0.18 (−0.45; 0.80)		0.00 (−0.69; 0.69)
0.21 (−0.22; 0.63)	0.15 (−0.23; 0.53)	0.21 (−0.26; 0.68)	−0.34 (−1.19; 0.51)	0.04 (−0.39; 0.48)	**Home MIT**		0.28 (−0.03; 0.60)
0.11 (−0.50; 0.72)	0.05 (−0.52; 0.63)	0.11 (−0.35; 0.57)	−0.44 (−1.39; 0.52)	−0.06 (−0.68; 0.56)	−0.10 (−0.67; 0.47)	**Home MIT + R**	0.14 (−0.63; 0.90)
**0.41 (0.05; 0.77)**	**0.36 (0.05; 0.67)**	0.41 (−0.03; 0.86)	−0.13 (−0.93; 0.66)	0.25 (−0.19; 0.68)	0.20 (−0.08; 0.49)	0.30 (−0.23; 0.83)	**nEX**

Health-related quality of life—mental component score							
**In-person HIIT**	0.06 (−0.16; 0.27)						0.00 (−0.54; 0.54)
−0.00 (−0.21; 0.20)	**In-person MIT**	−0.36 (−1.22; 0.50)			0.48 (−0.06; 1.02)		**0.57 (0.25; 0.89)**
0.09 (−0.39; 0.58)	0.10 (−0.36; 0.55)	**In-person MIT + R**		−0.16 (−0.92; 0.59)	0.34 (−0.48; 1.16)	0.12 (−0.32; 0.57)	0.17 (−0.69; 1.03)
**−0.82 (−1.49; −0.14)**	**−0.81 (−1.47; −0.16)**	**−0.91 (−1.64; −0.17)**	**In-person STBT**				**1.19 (0.58; 1.79)**
0.29 (−0.19; 0.78)	0.30 (−0.16; 0.76)	0.20 (−0.28; 0.69)	**1.11 (0.39; 1.84)**	**Home HIIT**	0.00 (−0.53; 0.53)		−0.25 (−0.92; 0.42)
0.27 (−0.09; 0.64)	0.28 (−0.05; 0.60)	0.18 (−0.26; 0.62)	**1.09 (0.44; 1.74)**	−0.02 (−0.41; 0.37)	**Home MIT**		0.16 (−0.11; 0.43)
0.22 (−0.32; 0.77)	0.23 (−0.29; 0.75)	0.13 (−0.26; 0.53)	**1.04 (0.27; 1.81)**	−0.07 (−0.63; 0.49)	−0.05 (−0.56; 0.46)	**Home MIT + R**	0.12 (−0.56; 0.81)
**0.37 (0.07; 0.67)**	**0.37 (0.11; 0.64)**	0.28 (−0.14; 0.70)	**1.19 (0.58; 1.79)**	0.07 (−0.33; 0.47)	0.10 (−0.15; 0.34)	0.15 (−0.33; 0.62)	**nEX**

Upper right triangle presents the pooled mean differences from direct comparisons and the lower left triangle pooled mean differences from the network meta-analysis. Columns are relative to the rows. Statistically significant differences are highlighted in bold values. Positive differences indicate an improvement in quality of life.

HIIT, high-intensity interval training; HIIT + R, high-intensity interval training plus resistance training; MIT, moderate-intensity training; MIT + R, moderate-intensity training plus resistance training; STBT, stretching-toning-balance training.

In-person HIIT (SMD 0.41; 95% CI 0.05–0.77) and in-person MIT (SMD 0.36; 95% CI 0.05; 0.67) were significantly associated with improvements in the PCS compared with no exercise (*Figure 3* and *Table 3*). In-person STBT (SMD 1.19; 95% CI 0.58; 1.79), in-person MIT (SMD 0.37; 95% CI 0.11; 0.64), and in-person HIIT (SMD 0.37; 95% CI 0.07; 0.67) were significantly associated with improvements in the MCS (*Figure 3* and *Table 3*). In-person STBT was solely based on one comparison (that was rated as high risk of bias, see Supplementary data online, *Figure S2*), whereas in-person HIIT and MIT were based on multiple comparisons and are therefore more robust estimates. No differences were found between HIIT and MIT in the improvement of the PCS and MCS, either in-person and home-based (*Table 3*).

All pairwise comparisons for the different HR-QoL outcomes are shown in Supplementary data online, *Figures S4*–*S6*. We found no global inconsistency using the random-effects model for the overall score of HR-QoL, PCS, and MCS, but local inconsistency was presented for some comparisons (see Supplementary data online, *Figures S7*–*S9*). The funnel plots included both positive and negative mean differences (see Supplementary data online, *Figure S10*).

### Different types of exercise and depressive and anxiety symptoms

Only pairwise comparisons (i.e. conventional meta-analysis and not network meta-analysis) were performed for depression and anxiety due to star-shaped networks (see Supplementary data online, *Figures S11* and *S12*). No statistically significant improvements for depression and anxiety were found for different types of exercise.

### In-person vs. home-based exercise

**Figure 4:**
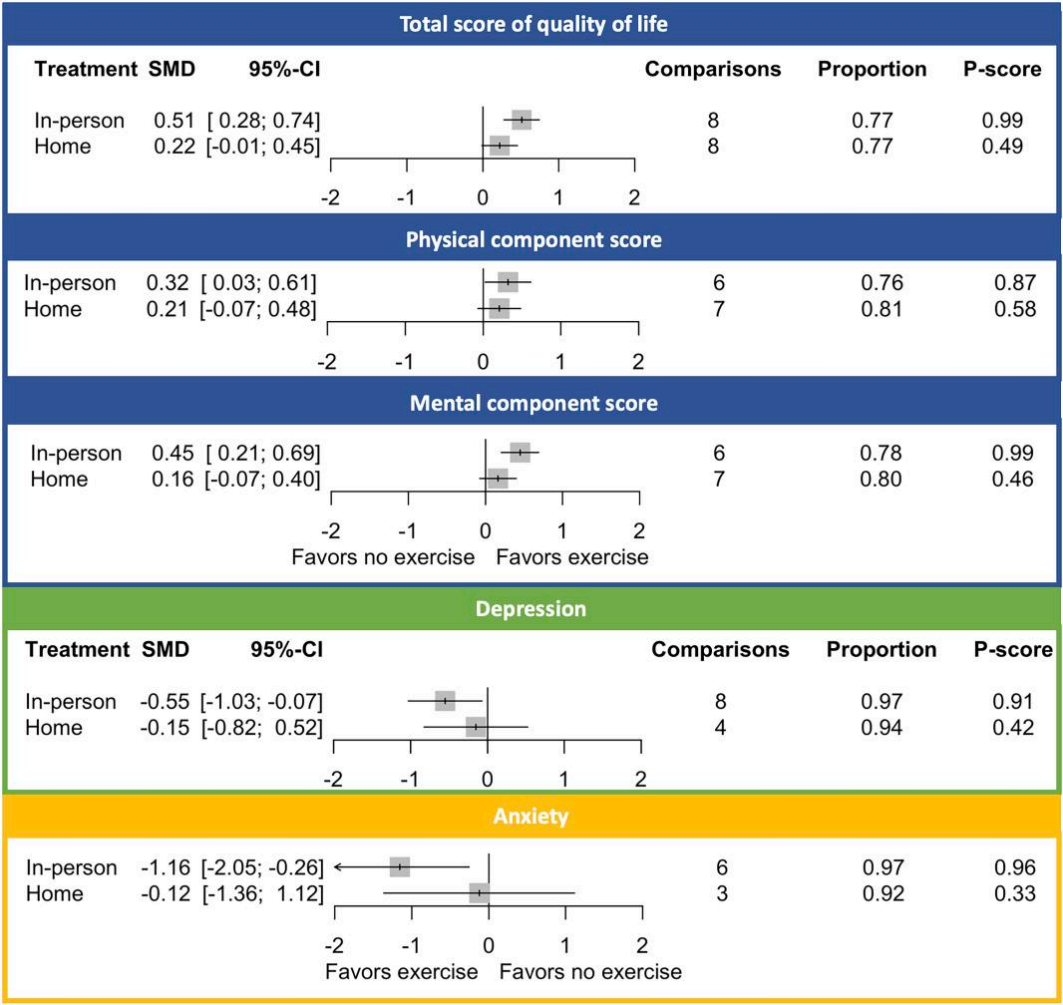
Health-related quality of life estimates for total, physical, and mental component scores for different settings of exercise training compared with no exercise using the network meta-analysis. The comparisons are the number of studies included in the analysis. The proportion refers to the amount of direct evidence that was used to determine the effect estimates. *P*-scores were scaled from 0 (worst) to 1 (best). For quality of life, positive differences indicate an improvement, whereas for depression and anxiety, negative differences indicate a reduction in symptoms. SMD, standardized mean difference; 95% CI, 95% confidence interval

In-person exercise was associated with significant improvements of HR-QoL, depression, and anxiety compared with no exercise (*Figure 4*, *Table 4*, and *Structured Graphical Abstract*). A smaller and non-significant effect of home exercise on all outcomes was found. All pairwise comparisons are shown in Supplementary data online, *Figures S13*–*S17*. There was no evidence for global or local inconsistency (see Supplementary data online, *Figures S18*–*S22*).

**Table 4:** League table with pooled standardized mean difference for different settings of exercise training and health-related quality of life, depression, and anxiety

Health-related quality of life—total score		
**In-person**	0.17 (−0.15; 0.49)	**0.60 (0.33; 0.87)**
**0.29 (0.04; 0.55)**	**Home**	0.13 (−0.14; 0.39)
**0.51 (0.28; 0.74)**	0.22 (−0.01; 0.45)	**nEX**
**Health-related quality of life—physical component score**		
**In-person**	0.16 (−0.22; 0.54)	0.32 (−0.01; 0.66)
0.11 (−0.20; 0.42)	**Home**	0.22 (−0.08; 0.52)
**0.32 (0.03; 0.61)**	0.21 (−0.07; 0.48)	**nEX**
**Health-related quality of life—mental component score**		
**In-person**	0.21 (−0.11; 0.54)	**0.53 (0.25; 0.81)**
**0.29 (0.02; 0.55)**	**Home**	0.10 (−0.16; 0.37)
**0.45 (0.21; 0.69)**	0.16 (−0.07; 0.40)	**nEX**
**Depression**		
**In-person**	−0.38 (−1.81; 1.04)	**−0.55 (−1.03; −0.07)**
−0.40 (−1.18; 0.38)	**Home**	−0.15 (−0.84; 0.55)
**−0.55 (−1.03; −0.07)**	−0.15 (−0.82; 0.52)	**nEX**
**Anxiety**		
**In-person**	−0.58 (−2.79; 1.64)	**−1.18 (−2.09; −0.27)**
−1.03 (−2.45; 0.39)	**Home**	−0.02 (−1.31; 1.27)
**−1.16 (−2.05; −0.26)**	−0.12 (−1.36; 1.12)	**nEX**

Upper right triangle presents the pooled mean differences from direct comparisons and the lower left triangle pooled mean differences from the network meta-analysis. Columns are relative to the rows. Statistically significant differences are highlighted in bold values. For quality of life, positive differences indicate an improvement, whereas for depression and anxiety, negative differences indicate a reduction in symptoms.

### Additional analyses

Secondary analyses examining the effect of the intervention duration and total exercise volume of the intervention on HR-QoL showed similar effect estimates between longer vs. shorter intervention duration and between larger vs. smaller total training volumes, in which all were in favour of in-person exercise. Except for the PCS when comparing in-person exercise >12 weeks, in-person >24 h, and in-person ≤24 h with no exercise, no significant effects were found (see Supplementary data online, *Tables S3* and *S4*). Sensitivity analyses using a conservative correlation (0.5) and excluding studies with a high risk of bias resulted in similar effect estimates compared with our main analyses (see Supplementary data online, *Tables S1* and *S2*).

## Discussion

This systematic review and network meta-analysis showed that most research assessing the effects of exercise on brain-related outcomes in CAD patients focused on HR-QoL, depression and anxiety, while there was a lack of studies assessing other dimensions of brain-related outcomes, such as brain structure/function, cognition or sleep. The network meta-analysis revealed that in-person exercise training was associated with improved HR-QoL and reduced depressive and anxiety symptoms, while the same type of exercise delivered at home had markedly and consistently smaller effect estimates for improving these outcomes. Furthermore, HIIT seems to be equally effective as MIT in improving these outcomes. Likewise, the intervention duration or volume of exercise training did not impact the outcomes. Overall, these findings suggest that exercise training, especially in-person programmes, could improve the HR-QoL, attenuate depressive symptoms, and reduce anxiety in patients with CAD. These findings were robust and persisted in our secondary and sensitivity analyses (i.e. excluding studies with high risk of bias).

### Effects of exercise on HR-QoL

HR-QoL is reduced in patients with CAD, especially after diagnosing CAD.^88,89^ The present meta-analysis indicates that in-person exercise training is associated with improved HR-QoL outcomes (i.e. overall, PCS, and MCS), independent of the type of exercise. This beneficial impact of exercise on HR-QoL is consistent with previous meta-analyses including other diseased populations, such as patients with heart failure,^90^ stroke,^91^ cancer,^92^ or Parkinson disease.^93^

We found that HIIT had a similar effect estimate as MIT for improving HR-QoL. This aligns with results of previous meta-analyses,^94,95^ except for one previous study in which HIIT exhibited greater improvement in PCS compared with MIT.^95^ Several types of exercise had only one comparison (e.g. HIIT + R and STBT), and no strong conclusions can be therefore drawn for these intervention types. Particularly interesting is the lack of studies evaluating the effects of resistance exercise alone, which might impact brain-related outcomes of CAD patients,^96,97^ and therefore needs more research in the future. Recent studies are developing exercise programmes combining both aerobic and resistance training, which aligns with the recommendations of the World Health Organization.^36^ Our network meta-analysis suggested that HIIT + R could be effective in improving HR-QoL and that MIT + R showed a tendency towards positive effects, although these were not statistically significant. One advantage of our network meta-analysis approach is the incorporation of indirect comparisons, enabling for the inclusion of more studies. However, these results should be interpreted with some caution as there is a large heterogeneity in the findings. This heterogeneity could be attributed to differences in the studies, such as characteristics of the exercise intervention (e.g. intensity continuum, diversity on the exercises included, such as different types of activities or equipment, outdoor or indoor) or CAD patient population (e.g. severity of disease, time after diagnosis, baseline mental status or medication use), which have been pointed to exert distinct impacts on the HR-QoL of patients with CAD.^88^ Our network meta-analysis is an important step in comparing different types of exercise, but future studies are needed to unravel the moderating effects of different factors (i.e. characteristics of exercise interventions and patient population) to move towards a more ‘personalized medicine’ approach to exercise prescription in CAD patients.

A major contribution of our study to the existing literature is the differential effect sizes of in-person vs. home-based exercise. These differences could be attributed to several factors, such as differences in adherence, better monitoring and live feedback-to-patients of face-to-face sessions, and/or social interaction. However, these factors are poorly and heterogeneously reported in the included studies. Interestingly, our results are contradictory to a previous systematic review^98^ in CVD patients. In this review, the authors concluded that both home- and centre-based CR provided similar improvements in HR-QoL. Potential explanations for the conflicting outcomes may relate to differences in the target population (all CVDs vs. only CAD in our study), interventions (CR vs. exercise in our study), number of included studies (17 vs. 30 in our study), and methodological approach (they did not run any meta-analysis *per se* on this outcome but used vote counting vs. network meta-analysis in our study). In addition, the authors decided that pooling data were too complex due to large variations in HR-QoL instruments. Nevertheless, we included standardized changes-from-baseline and ranked instruments to pool data and perform the network meta-analysis. Our network meta-analysis further allowed multiple comparisons using both direct and indirect effects, which resulted in a clear differential effect between exercise settings, i.e. two times larger effect in total HR-QoL and three times larger effect in MCS when comparing in-person vs. home-based.

### Effects of exercise on depressive and anxiety symptoms

In-person exercise interventions were associated with a positive effect on reducing depressive and anxiety symptoms among CAD patients, while home-based interventions did not yield significant improvements in both outcomes. Interestingly, the examination of various exercise types, yielded no statistically significant effects, and the same happened when classifying interventions based on their duration and total volume. Consistent with previous literature, our work holds the promise in ameliorating anxiety and depression symptoms in CAD patients with exercise.^99–101^ Most previous studies addressing depressive and anxiety symptoms on CAD have focused on multi-component CR programmes, and therefore, interpreting and comparing these findings becomes challenging since not all changes can be solely attributed to exercise.^98,100^ Furthermore, baseline levels of anxiety and depression may also impact the effects of exercise^100^ in which exercise might provide even bigger benefits in patients with more depressive and anxiety symptoms.

### Clinical implications

To enhance participation in CR programmes, home-based exercise interventions are becoming increasingly popular, and a previous systematic review had suggested that they can be equally effective as in-person exercise-based programmes.^98^ However, in the present updated and comprehensive systematic review, using data harmonization and advanced network meta-analysis techniques, we observed a clear differential effect, suggesting that in-person programmes are markedly superior in improving HR-QoL, depression, and anxiety in CAD patients. These findings have important clinical implications. While home-based interventions may be a promising (and probably necessary) strategy to encourage participation in exercise programmes, the present network meta-analysis suggests that the current literature raises concerns regarding its effectiveness in improving HR-QoL, depression, and anxiety and therefore opens venues for discussion on which features of home-based programmes could be optimized to become more effective.

### Strengths and limitations

The main strengths of our study are that we analysed the effects of different types, settings, duration, and volume (and indirectly intensity) of exercise on several brain-related dimensions and that we used a network meta-analysis approach enabling for both direct and indirect effect estimates. Furthermore, the search and selection process has been rigorously carried out, according to the most up-to-date guidelines and including many electronic databases to guarantee adequate and efficient coverage. Our network meta-analysis is based only on RCTs, which is the best study design available to investigate cause-effects.

Our study also has some limitations that must be addressed. First, the selection process has been restricted to studies examining the isolated effects of exercise, and we excluded grey literature, which might have impacted our results. Second, nearly half of the studies were rated with some concerns for the risk of bias and another 22% as high risk of bias. However, the sensitivity analyses excluding studies with a high risk of bias revealed similar effect estimates. To increase the quality of evidence, studies need to improve their reporting about the randomization process, study deviations, and (pre-) selected outcomes.^102^ Third, several types of exercise had only one comparison (e.g. HIIT + R and STBT), and no major conclusions can therefore be drawn about the effectiveness of these types of exercise. Future network meta-analyses including more studies should confirm our results for different types of exercise. Furthermore, some potential local inconsistencies were found, which could not be explained by the study characteristics. However, additional comparisons improving both direct and indirect evidence and under-reported factors, such as adherence, compliance and intensity, are important to improve the current study and need further investigation. Finally, we had significant heterogeneity in the analyses, which might be explained by variability in several key factors such as differences in characteristics of the patients and exercise interventions, as these factors could lead to different physiological adaptations. Furthermore, the included studies used a wide variety of instruments to assess brain-related outcomes. The use of different instruments forced us to include standardized changes-from-baseline, which could also introduce heterogeneity. Nevertheless, we standardized the change-from-baseline using pooled pre-SDs of studies using the same instruments.

## Conclusions

Our systematic review has identified that most of the current research on exercise brain-related outcomes in CAD patients is focused on HR-QoL, depression, and anxiety and highlights the need of studies assessing other dimensions of brain, such as brain structure/function, cognition, and sleep. Second, our network meta-analysis provided robust evidence supporting that in-person exercise interventions were associated with improved HR-QoL (including both the PCS and MCS) as well as reduced depressive and anxiety symptoms and should therefore be encouraged by health care providers. Furthermore, our study supports that the effectiveness of exercise reduces considerably when delivered by home-based interventions. Third, HIIT seems to be equally effective as MIT in improving these outcomes, suggesting that intensity might not impact these outcomes, yet needs further study. Future research should thoroughly investigate whether specific types or novel approaches in home-based interventions could prove more effective in enhancing brain-related outcomes than those utilized until the moment.

## Supplementary Material

ehae870_Supplementary_Data

## Declarations

### Data Availability

No data were generated. All extracted data and data processing scripts are available upon request to the corresponding authors.

### Pre-registered Clinical Trial Number

Study protocol was registered in PROSPERO (ID: CRD42023402569).
