# Supplementary material for: Exercise type and settings, quality of life, and mental health in coronary artery disease: a network meta-analysis
Source: Eur Heart J. 2025 Jan 15;46(23):2186–201. doi: 10.1093/eurheartj/ehae870 (PMC12167663; doi:10.1093/eurheartj/ehae870)
Supplement: ehae870_Supplementary_Data [file ehae870_supplementary_data.zip › Supplemental File 1. Methods_Search strategy.docx]

**Supplemental File 1. Methods_Search strategy**

**- Single-Line Search:**

(“coronary artery disease” OR “coronary heart disease” OR “ischemic heart disease” OR “heart disease”) AND (Exercise OR “physical activity” OR “musc* strength” OR “strength training” OR “strength exercise” OR “resistance training” OR “resistance exercise” OR “endurance training” OR “endurance exercise” OR “aerobic training” OR “aerobic exercise”) AND (Brain OR neur* OR cereb* OR “grey matter” OR “gray matter” OR “white matter” OR “frontal cortex” OR “prefrontal cortex” OR PFC OR parietal OR “temporal lobe” OR “occipital lobe” OR hippocamp* OR striat* OR accumbens OR caudate OR putamen OR “globus pallidus” OR thalam* OR subthalam* OR hypothalam* OR forebrain OR midbrain OR mesenceph* OR hindbrain OR diencephal* OR “brain stem” OR cognition OR cognitive OR memory OR “executive function*“ OR “problem solving“ OR “perceptual process*“ OR neurocognitive OR “mental flexibility“ OR “mental recall“ OR “inhibitory control“ OR “information processing” OR “executive control” OR “attentional control” OR Mental OR psychologic* OR psychosocial OR Emotion* OR “Well being” OR “Well-being” OR “quality of life” OR “life quality” OR “Self-esteem” OR “Self esteem” OR Happiness OR Mood OR Optimism OR Pessimism OR Anxiety OR Depress* OR “Life stress” OR “Psychologic stress” OR “stress management” OR sleep) AND (Trial OR “Intervention study” OR RCT OR crossover)

- **Pubmed:**

("coronary artery disease"[tiab] OR "coronary heart disease"[tiab] OR "ischemic heart disease"[tiab] OR "heart disease"[tiab]) AND (Exercise[tiab] OR "physical activity"[tiab] OR "musc* strength"[tiab] OR "strength training"[tiab] OR "strength exercise"[tiab] OR "resistance training"[tiab] OR "resistance exercise"[tiab] OR "endurance training"[tiab] OR "endurance exercise"[tiab] OR "aerobic training"[tiab] OR "aerobic exercise"[tiab]) AND (Brain[tiab] OR neur*[tiab] OR cereb*[tiab] OR "grey matter"[tiab] OR "gray matter"[tiab] OR "white matter"[tiab] OR "frontal cortex"[tiab] OR "prefrontal cortex"[tiab] OR PFC[tiab] OR parietal[tiab] OR "temporal lobe"[tiab] OR "occipital lobe"[tiab] OR hippocamp*[tiab] OR striat*[tiab] OR accumbens[tiab] OR caudate[tiab] OR putamen[tiab] OR "globus pallidus"[tiab] OR thalam*[tiab] OR subthalam*[tiab] OR hypothalam*[tiab] OR forebrain[tiab] OR midbrain[tiab] OR mesenceph*[tiab] OR hindbrain[tiab] OR diencephal*[tiab] OR "brain stem"[tiab] OR cognition[tiab] OR cognitive[tiab] OR memory[tiab] OR "executive function*"[tiab] OR "problem solving"[tiab] OR "perceptual process*"[tiab] OR neurocognitive[tiab] OR "mental flexibility"[tiab] OR "mental recall"[tiab] OR "inhibitory control"[tiab] OR "information processing"[tiab] OR "executive control"[tiab] OR "attentional control"[tiab] OR Mental[tiab] OR psychologic*[tiab] OR psychosocial[tiab] OR Emotion*[tiab] OR "Well being"[tiab] OR Well-being[tiab] OR "quality of life"[tiab] OR "life quality"[tiab] OR Self-esteem[tiab] OR "Self esteem"[tiab] OR Happiness[tiab] OR Mood[tiab] OR Optimism[tiab] OR Pessimism[tiab] OR Anxiety[tiab] OR Depress*[tiab] OR "Life stress*"[tiab] OR "Psychologic stress"[tiab] OR "stress management"[tiab] OR sleep[tiab]) AND (Trial[tiab] OR "Intervention study"[tiab] OR RCT[tiab] OR crossover[tiab])

**- WOS:**

*Search combining title + abstracts + keywords plus, and searching on ALL Databases

((TI=(("coronary artery disease" OR "coronary heart disease" OR "ischemic heart disease" OR "heart disease") AND (Exercise OR "physical activity" OR "musc* strength" OR "strength training" OR "strength exercise" OR "resistance training" OR "resistance exercise" OR "endurance training" OR "endurance exercise" OR "aerobic training" OR "aerobic exercise") AND (Brain OR neur* OR cereb* OR "grey matter" OR "gray matter" OR "white matter" OR "frontal cortex" OR "prefrontal cortex" OR PFC OR parietal OR "temporal lobe" OR "occipital lobe" OR hippocamp* OR striat* OR accumbens OR caudate OR putamen OR "globus pallidus" OR thalam* OR subthalam* OR hypothalam* OR forebrain OR midbrain OR mesenceph* OR hindbrain OR diencephal* OR "brain stem" OR cognition OR cognitive OR memory OR "executive function*" OR "problem solving" OR "perceptual process*" OR neurocognitive OR "mental flexibility" OR "mental recall" OR "inhibitory control" OR "information processing" OR "executive control" OR "attentional control" OR Mental OR psychologic* OR psychosocial OR Emotion* OR "Well being" OR Well-being OR "quality of life" OR "life quality" OR Self-esteem OR "Self esteem" OR Happiness OR Mood OR Optimism OR Pessimism OR Anxiety OR Depress* OR "Life stress*" OR "Psychologic stress" OR "stress management" OR sleep) AND (Trial OR "Intervention study" OR RCT OR crossover))) OR AB=(("coronary artery disease" OR "coronary heart disease" OR "ischemic heart disease" OR "heart disease") AND (Exercise OR "physical activity" OR "musc* strength" OR "strength training" OR "strength exercise" OR "resistance training" OR "resistance exercise" OR "endurance training" OR "endurance exercise" OR "aerobic training" OR "aerobic exercise") AND (Brain OR neur* OR cereb* OR "grey matter" OR "gray matter" OR "white matter" OR "frontal cortex" OR "prefrontal cortex" OR PFC OR parietal OR "temporal lobe" OR "occipital lobe" OR hippocamp* OR striat* OR accumbens OR caudate OR putamen OR "globus pallidus" OR thalam* OR subthalam* OR hypothalam* OR forebrain OR midbrain OR mesenceph* OR hindbrain OR diencephal* OR "brain stem" OR cognition OR cognitive OR memory OR "executive function*" OR "problem solving" OR "perceptual process*" OR neurocognitive OR "mental flexibility" OR "mental recall" OR "inhibitory control" OR "information processing" OR "executive control" OR "attentional control" OR Mental OR psychologic* OR psychosocial OR Emotion* OR "Well being" OR Well-being OR "quality of life" OR "life quality" OR Self-esteem OR "Self esteem" OR Happiness OR Mood OR Optimism OR Pessimism OR Anxiety OR Depress* OR "Life stress*" OR "Psychologic stress" OR "stress management" OR sleep) AND (Trial OR "Intervention study" OR RCT OR crossover))) OR KP=(("coronary artery disease" OR "coronary heart disease" OR "ischemic heart disease" OR "heart disease") AND (Exercise OR "physical activity" OR "musc* strength" OR "strength training" OR "strength exercise" OR "resistance training" OR "resistance exercise" OR "endurance training" OR "endurance exercise" OR "aerobic training" OR "aerobic exercise") AND (Brain OR neur* OR cereb* OR "grey matter" OR "gray matter" OR "white matter" OR "frontal cortex" OR "prefrontal cortex" OR PFC OR parietal OR "temporal lobe" OR "occipital lobe" OR hippocamp* OR striat* OR accumbens OR caudate OR putamen OR "globus pallidus" OR thalam* OR subthalam* OR hypothalam* OR forebrain OR midbrain OR mesenceph* OR hindbrain OR diencephal* OR "brain stem" OR cognition OR cognitive OR memory OR "executive function*" OR "problem solving" OR "perceptual process*" OR neurocognitive OR "mental flexibility" OR "mental recall" OR "inhibitory control" OR "information processing" OR "executive control" OR "attentional control" OR Mental OR psychologic* OR psychosocial OR Emotion* OR "Well being" OR Well-being OR "quality of life" OR "life quality" OR Self-esteem OR "Self esteem" OR Happiness OR Mood OR Optimism OR Pessimism OR Anxiety OR Depress* OR "Life stress*" OR "Psychologic stress" OR "stress management" OR sleep) AND (Trial OR "Intervention study" OR RCT OR crossover))

- **Scopus:**

(TITLE-ABS("coronary artery disease") OR TITLE-ABS("coronary heart disease") OR TITLE-ABS("ischemic heart disease") OR TITLE-ABS("heart disease")) AND (TITLE-ABS(Exercise) OR TITLE-ABS("physical activity") OR TITLE-ABS("musc* strength") OR TITLE-ABS("strength training") OR TITLE-ABS("strength exercise") OR TITLE-ABS("resistance training") OR TITLE-ABS("resistance exercise") OR TITLE-ABS("endurance training") OR TITLE-ABS("endurance exercise") OR TITLE-ABS("aerobic training") OR TITLE-ABS("aerobic exercise")) AND (TITLE-ABS(Brain) OR TITLE-ABS(neur*) OR TITLE-ABS(cereb*) OR TITLE-ABS("grey matter") OR TITLE-ABS("gray matter") OR TITLE-ABS("white matter") OR TITLE-ABS("frontal cortex") OR TITLE-ABS("prefrontal cortex") OR TITLE-ABS(PFC) OR TITLE-ABS(parietal) OR TITLE-ABS("temporal lobe") OR TITLE-ABS("occipital lobe") OR TITLE-ABS(hippocamp*) OR TITLE-ABS(striat*) OR TITLE-ABS(accumbens) OR TITLE-ABS(caudate) OR TITLE-ABS(putamen) OR TITLE-ABS("globus pallidus") OR TITLE-ABS(thalam*) OR TITLE-ABS(subthalam*) OR TITLE-ABS(hypothalam*) OR TITLE-ABS(forebrain) OR TITLE-ABS(midbrain) OR TITLE-ABS(mesenceph*) OR TITLE-ABS(hindbrain) OR TITLE-ABS(diencephal*) OR TITLE-ABS("brain stem") OR TITLE-ABS(cognition) OR TITLE-ABS(cognitive) OR TITLE-ABS(memory) OR TITLE-ABS("executive function*") OR TITLE-ABS("problem solving") OR TITLE-ABS("perceptual process*") OR TITLE-ABS(neurocognitive) OR TITLE-ABS("mental flexibility") OR TITLE-ABS("mental recall") OR TITLE-ABS("inhibitory control") OR TITLE-ABS("information processing") OR TITLE-ABS("executive control") OR TITLE-ABS("attentional control") OR TITLE-ABS(Mental) OR TITLE-ABS(psychologic*) OR TITLE-ABS(psychosocial) OR TITLE-ABS(Emotion*) OR TITLE-ABS("Well being") OR TITLE-ABS(Well-being) OR TITLE-ABS("quality of life") OR TITLE-ABS("life quality") OR TITLE-ABS(Self-esteem) OR TITLE-ABS("Self esteem") OR TITLE-ABS(Happiness) OR TITLE-ABS(Mood) OR TITLE-ABS(Optimism) OR TITLE-ABS(Pessimism) OR TITLE-ABS(Anxiety) OR TITLE-ABS(Depress*) OR TITLE-ABS("Life stress*") OR TITLE-ABS("Psychologic stress") OR TITLE-ABS("stress management") OR TITLE-ABS(sleep)) AND (TITLE-ABS(Trial) OR TITLE-ABS("Intervention study") OR TITLE-ABS(RCT) OR TITLE-ABS(crossover))

- **Cochrane:**

("coronary artery disease":ti,ab OR "coronary heart disease":ti,ab OR "ischemic heart disease":ti,ab OR "heart disease":ti,ab) AND (Exercise:ti,ab OR "physical activity":ti,ab OR (musc* NEXT "strength"):ti,ab OR "strength training":ti,ab OR "strength exercise":ti,ab OR "resistance training":ti,ab OR "resistance exercise":ti,ab OR "endurance training":ti,ab OR "endurance exercise":ti,ab OR "aerobic training":ti,ab OR "aerobic exercise":ti,ab) AND (Brain:ti,ab OR neur*:ti,ab OR cereb*:ti,ab OR "grey matter":ti,ab OR "gray matter":ti,ab OR "white matter":ti,ab OR "frontal cortex":ti,ab OR "prefrontal cortex":ti,ab OR PFC:ti,ab OR parietal:ti,ab OR "temporal lobe":ti,ab OR "occipital lobe":ti,ab OR hippocamp*:ti,ab OR striat*:ti,ab OR accumbens:ti,ab OR caudate:ti,ab OR putamen:ti,ab OR "globus pallidus":ti,ab OR thalam*:ti,ab OR subthalam*:ti,ab OR hypothalam*:ti,ab OR forebrain:ti,ab OR midbrain:ti,ab OR mesenceph*:ti,ab OR hindbrain:ti,ab OR diencephal*:ti,ab OR "brain stem":ti,ab OR cognition:ti,ab OR cognitive:ti,ab OR memory:ti,ab OR ("executive" NEXT function*):ti,ab OR "problem solving":ti,ab OR ("perceptual" NEXT process*):ti,ab OR neurocognitive:ti,ab OR "mental flexibility":ti,ab OR "mental recall":ti,ab OR "inhibitory control":ti,ab OR "information processing":ti,ab OR "executive control":ti,ab OR "attentional control":ti,ab OR Mental:ti,ab OR psychologic*:ti,ab OR psychosocial:ti,ab OR Emotion*:ti,ab OR "Well being":ti,ab OR Well-being:ti,ab OR "quality of life":ti,ab OR "life quality":ti,ab OR Self-esteem:ti,ab OR "Self esteem":ti,ab OR Happiness:ti,ab OR Mood:ti,ab OR Optimism:ti,ab OR Pessimism:ti,ab OR Anxiety:ti,ab OR Depress*:ti,ab OR "Life stress":ti,ab OR "Psychologic stress":ti,ab OR "stress management":ti,ab OR sleep:ti,ab) AND (Trial:ti,ab OR "Intervention study":ti,ab OR RCT:ti,ab OR crossover:ti,ab)

- **SportDiscuss**

TI ( “coronary artery disease” OR “coronary heart disease” OR “ischemic heart disease” OR “heart disease” ) AND TI ( Exercise OR “physical activity” OR “musc* strength” OR “strength training” OR “strength exercise” OR “resistance training” OR “resistance exercise” OR “endurance training” OR “endurance exercise” OR “aerobic training” OR “aerobic exercise” ) AND TI ( Brain OR neur* OR cereb* OR “grey matter” OR “gray matter” OR “white matter” OR “frontal cortex” OR “prefrontal cortex” OR PFC OR parietal OR “temporal lobe” OR “occipital lobe” OR hippocamp* OR striat* OR accumbens OR caudate OR putamen OR “globus pallidus” OR thalam* OR subthalam* OR hypothalam* OR forebrain OR midbrain OR mesenceph* OR hindbrain OR diencephal* OR “brain stem” OR cognition OR cognitive OR memory OR “executive function*“ OR “problem solving“ OR “perceptual process*“ OR neurocognitive OR “mental flexibility“ OR “mental recall“ OR “inhibitory control“ OR “information processing” OR “executive control” OR “attentional control” OR Mental OR psychologic* OR psychosocial OR Emotion* OR “Well being” OR “Well-being” OR “quality of life” OR “life quality” OR “Self-esteem” OR “Self esteem” OR Happiness OR Mood OR Optimism OR Pessimism OR Anxiety OR Depress* OR “Life stress*” OR “Psychologic stress” OR “stress management” OR sleep ) AND TI ( Trial OR “Intervention study” OR RCT OR crossover ) OR AB ( “coronary artery disease” OR “coronary heart disease” OR “ischemic heart disease” OR “heart disease” ) AND AB ( Exercise OR “physical activity” OR “musc* strength” OR “strength training” OR “strength exercise” OR “resistance training” OR “resistance exercise” OR “endurance training” OR “endurance exercise” OR “aerobic training” OR “aerobic exercise” ) AND AB ( Brain OR neur* OR cereb* OR “grey matter” OR “gray matter” OR “white matter” OR “frontal cortex” OR “prefrontal cortex” OR PFC OR parietal OR “temporal lobe” OR “occipital lobe” OR hippocamp* OR striat* OR accumbens OR caudate OR putamen OR “globus pallidus” OR thalam* OR subthalam* OR hypothalam* OR forebrain OR midbrain OR mesenceph* OR hindbrain OR diencephal* OR “brain stem” OR cognition OR cognitive OR memory OR “executive function*“ OR “problem solving“ OR “perceptual process*“ OR neurocognitive OR “mental flexibility“ OR “mental recall“ OR “inhibitory control“ OR “information processing” OR “executive control” OR “attentional control” OR Mental OR psychologic* OR psychosocial OR Emotion* OR “Well being” OR “Well-being” OR “quality of life” OR “life quality” OR “Self-esteem” OR “Self esteem” OR Happiness OR Mood OR Optimism OR Pessimism OR Anxiety OR Depress* OR “Life stress*” OR “Psychologic stress” OR “stress management” OR sleep ) AND AB ( Trial OR “Intervention study” OR RCT OR crossover )

- **PsycInfo**

tiab(“coronary artery disease” OR “coronary heart disease” OR “ischemic heart disease” OR “heart disease”) AND tiab(Exercise OR “physical activity” OR “musc* strength” OR “strength training” OR “strength exercise” OR “resistance training” OR “resistance exercise” OR “endurance training” OR “endurance exercise” OR “aerobic training” OR “aerobic exercise”) AND tiab(Brain OR neur* OR cereb* OR “grey matter” OR “gray matter” OR “white matter” OR “frontal cortex” OR “prefrontal cortex” OR PFC OR parietal OR “temporal lobe” OR “occipital lobe” OR hippocamp* OR striat* OR accumbens OR caudate OR putamen OR “globus pallidus” OR thalam* OR subthalam* OR hypothalam* OR forebrain OR midbrain OR mesenceph* OR hindbrain OR diencephal* OR “brain stem” OR cognition OR cognitive OR memory OR “executive function*“ OR “problem solving“ OR “perceptual process*“ OR neurocognitive OR “mental flexibility“ OR “mental recall“ OR “inhibitory control“ OR “information processing” OR “executive control” OR “attentional control” OR Mental OR psychologic* OR psychosocial OR Emotion* OR “Well being” OR “Well-being” OR “quality of life” OR “life quality” OR “Self-esteem” OR “Self esteem” OR Happiness OR Mood OR Optimism OR Pessimism OR Anxiety OR Depress* OR “Life stress*” OR “Psychologic stress” OR “stress management” OR sleep) AND tiab(Trial OR “Intervention study” OR RCT OR crossover)

- **EMBASE:**

#1 ‘coronary artery disease’.ti,ab. OR ‘coronary heart disease’.ti,ab. OR ‘ischemic heart disease’.ti,ab. OR ‘heart disease’.ti,ab.

#2 Exercise.ti,ab. OR ‘physical activity’.ti,ab. OR ‘musc* strength’.ti,ab. OR ‘strength training’.ti,ab. OR ‘strength exercise’.ti,ab. OR ‘resistance training’.ti,ab. OR ‘resistance exercise’.ti,ab. OR ‘endurance training’.ti,ab. OR ‘endurance exercise’.ti,ab. OR ‘aerobic training’.ti,ab. OR ‘aerobic exercise’.ti,ab.

#3 Brain.ti,ab. OR neur*.ti,ab. OR cereb*.ti,ab. OR ‘grey matter’.ti,ab. OR ‘gray matter’.ti,ab. OR ‘white matter’.ti,ab. OR ‘frontal cortex’.ti,ab. OR ‘prefrontal cortex’.ti,ab. OR PFC.ti,ab. OR parietal.ti,ab. OR ‘temporal lobe’.ti,ab. OR ‘occipital lobe’.ti,ab. OR hippocamp*.ti,ab. OR striat*.ti,ab. OR accumbens.ti,ab. OR caudate.ti,ab. OR putamen.ti,ab. OR ‘globus pallidus’.ti,ab. OR thalam*.ti,ab. OR subthalam*.ti,ab. OR hypothalam*.ti,ab. OR forebrain.ti,ab. OR midbrain.ti,ab. OR mesenceph*.ti,ab. OR hindbrain.ti,ab. OR diencephal*.ti,ab. OR ‘brain stem’.ti,ab. OR cognition.ti,ab. OR cognitive.ti,ab. OR memory.ti,ab. OR ‘executive function*’.ti,ab. OR ‘problem solving’.ti,ab. OR ‘perceptual process*’.ti,ab. OR neurocognitive.ti,ab. OR ‘mental flexibility’.ti,ab. OR ‘mental recall’.ti,ab. OR ‘inhibitory control’.ti,ab. OR ‘information processing’.ti,ab. OR ‘executive control’.ti,ab. OR ‘attentional control’.ti,ab. OR Mental.ti,ab. OR psychologic*.ti,ab. OR psychosocial.ti,ab. OR Emotion*.ti,ab. OR ‘Well being’.ti,ab. OR ‘Well-being’.ti,ab. OR ‘quality of life’.ti,ab. OR ‘life quality’.ti,ab. OR ‘Self-esteem’.ti,ab. OR ‘Self esteem’.ti,ab. OR Happiness.ti,ab. OR Mood.ti,ab. OR Optimism.ti,ab. OR Pessimism.ti,ab. OR Anxiety.ti,ab. OR Depress*.ti,ab. OR ‘Life stress*’.ti,ab. OR ‘Psychologic stress’.ti,ab. OR ‘stress management’.ti,ab. OR sleep.ti,ab.

#4 Trial.ti,ab. OR Intervention study.ti,ab. OR RCT.ti,ab. OR crossover.ti,ab.

#1 AND #2 AND #3 AND #4

- **Google scholar** (sort by relevance)

(“coronary artery disease”|“coronary heart disease”|“ischemic heart disease”) AND (Exercise|“physical activity”|“resistance training”|“aerobic training”) AND (Brain|cereb*|cognit*|mental|depress*|“quality of life”) AND Trial
