# Supplementary material for: Exercise type and settings, quality of life, and mental health in coronary artery disease: a network meta-analysis
Source: Eur Heart J. 2025 Jan 15;46(23):2186–201. doi: 10.1093/eurheartj/ehae870 (PMC12167663; doi:10.1093/eurheartj/ehae870)
Supplement: ehae870_Supplementary_Data [file ehae870_supplementary_data.zip › Supplemental File 3. Tables figures_part 1_R1_clean.docx]

**Supplemental Table 1.** League table with pooled SMD for different exercise types and health-related quality of life; total, physical and mental component scores using a conservative correlation (R 0.5).

| **Health-related quality of life - Total score** | | | | | | | | | | | | | | | |
| --- | --- | --- | --- | --- | --- | --- | --- | --- | --- | --- | --- | --- | --- | --- | --- |
| **In-person HIIT** | . | | 0.03  (-0.17; 0.24) | | . | | . | | . | | . | | . | | 0.21  (-0.34; 0.76) |
| -1.11  (-1.90; -0.31) | **In-person HIIT + R** | | . | | . | | . | | . | | . | | . | | **1.53**  **(0.79; 2.28)** |
| 0.00  (-0.19; 0.20) | **1.11**  **(0.33; 1.89)** | | **In-person MIT** | | -0.36  (-1.18; 0.45) | | . | | . | | 0.31  (-0.07; 0.69) | | . | | **0.57**  **(0.29; 0.86)** |
| 0.13  (-0.35; 0.61) | **1.24**  **(0.37; 2.10)** | | 0.13  (-0.32; 0.58) | | **In-person MIT + R** | | . | | -0.09  (-0.85; 0.67) | | 0.25  (-0.66; 1.16) | | 0.05  (-0.40; 0.50) | | -0.03  (-1.02; 0.95) |
| -0.13  (-0.85; 0.60) | 0.98  (-0.02; 1.98) | | -0.13  (-0.84; 0.58) | | -0.25  (-1.05; 0.54) | | **In-person STBT** | | . | | . | | . | | 0.55  (-0.11; 1.22) |
| 0.38  (-0.09; 0.84) | **1.48**  **(0.64; 2.33)** | | 0.37  (-0.06; 0.80) | | 0.25  (-0.24; 0.74) | | 0.50  (-0.27; 1.28) | | **Home HIIT** | | -0.11  -0.64; 0.42) | | . | | -0.18  (-0.84; 0.48) |
| 0.28  (-0.04; 0.60) | **1.39**  **(0.61; 2.16)** | | **0.28**  **(0.01; 0.54)** | | 0.15  (-0.29; 0.60) | | 0.41  (-0.30; 1.11) | | -0.10  (-0.48; 0.29) | | **Home MIT** | | . | | 0.16  (-0.08; 0.41) |
| 0.19  (-0.36; 0.74) | **1.30**  **(0.40; 2.19)** | | 0.19  (-0.34; 0.71) | | 0.06  (-0.34; 0.46) | | 0.31  (-0.52; 1.15) | | -0.19  (-0.76; 0.39) | | -0.09  (-0.61; 0.43) | | **Home MIT + R** | | 0.22  (-0.52; 0.96) |
| **0.43**  **(0.15; 0.71)** | **1.53**  **(0.79; 2.28)** | | **0.42**  **(0.20; 0.65)** | | 0.30  (-0.13; 0.73) | | 0.55  (-0.11; 1.22) | | 0.05  (-0.34; 0.45) | | 0.15  (-0.07; 0.36) | | 0.24  (-0.26; 0.74) | | **nEX** |
| **Health-related quality of life - Physical component score** | | | | | | | | | | | | | | | |
| **In-person HIIT** | | 0.04  (-0.19; 0.28) | | . | | . | | . | | . | | . | | 0.47  (-0.17; 1.11) | |
| 0.05  (-0.17; 0.28) | | **In-person MIT** | | -0.37  (-1.13; 0.39) | | . | | . | | 0.34  (-0.28; 0.95) | | . | | **0.39**  **(0.02; 0.76)** | |
| -0.02  (-0.53; 0.49) | | -0.07  (-0.55; 0.40) | | **In-person MIT + R** | | . | | 0.07  (-0.64; 0.78) | | 0.37  (-0.64; 1.39) | | 0.05  (-0.47; 0.56) | | -0.03  (-1.20; 1.13) | |
| 0.53  (-0.36; 1.41) | | 0.47  (-0.40; 1.34) | | 0.54  (-0.39; 1.48) | | **In-person STBT** | | . | | . | | . | | -0.13  (-0.95; 0.68) | |
| 0.15  (-0.36; 0.66) | | 0.09  (-0.38; 0.57) | | 0.17  (-0.32; 0.66) | | -0.38  (-1.29; 0.53) | | **Home HIIT** | | 0.18  (-0.39; 0.75) | | . | | 0.00  (-0.64; 0.64) | |
| 0.19  (-0.22; 0.60) | | 0.14  (-0.23; 0.51) | | 0.21  (-0.26; 0.69) | | -0.33  (-1.19; 0.53) | | 0.04  (-0.36; 0.45) | | **Home MIT** | | . | | 0.29  (-0.01; 0.59) | |
| 0.10  (-0.51; 0.70) | | 0.04  (-0.53; 0.62) | | 0.12  (-0.34; 0.57) | | -0.43  (-1.40; 0.55) | | -0.05  (-0.65; 0.55) | | -0.10  (-0.67; 0.47) | | **Home MIT + R** | | 0.14  (-0.66; 0.93) | |
| **0.39**  **(0.05; 0.73)** | | **0.34**  **(0.04; 0.64)** | | 0.41  (-0.04; 0.86) | | -0.13  (-0.95; 0.68) | | 0.24  (-0.16; 0.65) | | 0.20  (-0.08; 0.47) | | 0.30  (-0.24; 0.83) | | **nEX** | |
| **Health-related quality of life - Mental component score** | | | | | | | | | | | | | | | |
| **In-person HIIT** | | 0.06  (-0.16; 0.28) | | . | | . | | . | | . | | . | | 0.00  (-0.53; 0.53) | |
| -0.00  (-0.21; 0.20) | | **In-person MIT** | | -0.36  (-1.30; 0.58) | | . | | . | | 0.48  (-0.05; 1.01) | | . | | **0.56**  **(0.25; 0.87)** | |
| 0.13  (-0.38; 0.63) | | 0.13  (-0.35; 0.61) | | **In-person MIT + R** | | . | | -0.16  (-0.90; 0.57) | | 0.34  (-0.58; 1.26) | | 0.10  (-0.34; 0.53) | | 0.17  (-0.79; 1.13) | |
| -0.82  (-1.51; -0.14) | | -0.82  (-1.49; -0.15) | | -0.95  (-1.71; -0.19) | | **In-person STBT** | | . | | . | | . | | **1.19**  **(0.57; 1.81)** | |
| 0.31  (-0.17; 0.78) | | 0.31  (-0.13; 0.75) | | 0.18  (-0.31; 0.67) | | 1.13  (0.40; 1.86) | | **Home HIIT** | | 0.00  (-0.50; 0.50) | | . | | -0.25  (-0.90; 0.40) | |
| 0.28  (-0.08; 0.64) | | 0.28  (-0.04; 0.60) | | 0.15  (-0.31; 0.61) | | 1.10  (0.44; 1.76) | | -0.03  (-0.40; 0.35) | | **Home MIT** | | . | | 0.16  (-0.11; 0.42) | |
| 0.23  (-0.33; 0.79) | | 0.23  (-0.31; 0.77) | | 0.10  (-0.29; 0.49) | | 1.05  (0.26; 1.84) | | -0.08  (-0.64; 0.48) | | -0.05  (-0.58; 0.47) | | **Home MIT + R** | | 0.12  (-0.61; 0.86) | |
| **0.37**  **(0.07; 0.66)** | | **0.37**  **(0.11; 0.63)** | | 0.24  (-0.20; 0.68) | | **1.19**  **(0.57; 1.81)** | | 0.06  (-0.33; 0.45) | | 0.09  (-0.16; 0.33) | | 0.14  (-0.36; 0.64) | | **nEX** | |
| Upper right triangle presents the pooled mean differences from direct comparisons and the lower left triangle pooled mean differences from the network meta-analysis. Columns are relative to the rows. Positive differences indicate an improvement in quality of life. | | | | | | | | | | | | | | | |

.

**Supplemental Table 2.** League table with pooled SMD for different exercise types and health-related quality of life; total, physical and mental component score excluding studies with a high risk of bias.

| **Health-related quality of life - Total score** | | | | | | |
| --- | --- | --- | --- | --- | --- | --- |
| **In-person HIIT** | 0.05  (-0.19; 0.29) | .  . | . | . | . | 0.21  (-0.37; 0.79) |
| 0.01  (-0.21; 0.23) | **In-person MIT**. | .  . | . | 0.31  (-0.10; 0.71) | . | **0.60**  **(0.30; 0.91)** |
| 0.37  (-0.20; 0.95) | 0.37  (-0.18; 0.91) | **In-person MIT + R**. | -0.09  (-0.89; 0.71) | 0.25  (-0.59; 1.09) | 0.02  (-0.55; 0.60) | -0.03  (-0.92; 0.86) |
| 0.49  (-0.02; 1.00) | **0.48**  **(0.00; 0.96)** | 0.11  (-0.43; 0.66) | **Home HIIT** | -0.11  (-0.69; 0.48) | . | -0.18  (-0.89; 0.52) |
| 0.38  (0.02; 0.74) | **0.37**  **(0.06; 0.68)** | 0.00  (-0.51; 0.52) | -0.11  (-0.54; 0.32) | **Home MIT** | . | 0.09  (-0.22; 0.41) |
| 0.34 (  -0.27; 0.96) | 0.33  (-0.26; 0.92) | -0.03  (-0.52; 0.46) | -0.15  (-0.78; 0.48) | -0.04  (-0.61; 0.54) | **Home MIT + R** | 0.22  (-0.50; 0.93) |
| **0.47**  **(0.17; 0.78)** | **0.46**  **(0.21; 0.72)** | 0.10  (-0.41; 0.60) | -0.02  (-0.45; 0.42) | 0.09  (-0.17; 0.36) | 0.13  (-0.41; 0.67) | **nEX** |
| **Quality of life - Physical component score** | | | | | | |
| **In-person HIIT** | 0.06  (-0.22; 0.34) | . | . | . | . | 0.47  (-0.24; 1.18) |
| 0.06  (-0.21; 0.33) | **In-person MIT** | . | . | 0.34  (-0.36; 1.04) | . | **0.44**  **(0.02; 0.86)** |
| 0.22  (-0.47; 0.91) | 0.16  (-0.51; 0.82) | **In-person MIT + R** | 0.07  (-0.73; 0.87) | 0.37  (-0.59; 1.34) | 0.11  (-0.58; 0.81) | -0.03  (-1.11; 1.04) |
| 0.31  (-0.31; 0.93) | 0.25  (-0.34; 0.83) | 0.09  (-0.49; 0.67) | **Home HIIT** | 0.18  (-0.50; 0.86) | . | 0.00  (-0.74; 0.74) |
| 0.37  (-0.15; 0.89) | 0.31  (-0.17; 0.79) | 0.16  (-0.44; 0.75) | 0.06  (-0.43; 0.55) | **Home MIT** | . | 0.20  (-0.24; 0.65) |
| 0.33  (-0.41; 1.06) | 0.26  (-0.45; 0.97) | 0.11  (-0.47; 0.69) | 0.02  (-0.69; 0.72) | -0.05  (-0.73; 0.64) | **Home MIT + R** | 0.14  (-0.68; 0.95) |
| **0.46**  **(0.05; 0.86)** | **0.40**  **(0.04; 0.76)** | 0.24  (-0.33; 0.82) | 0.15  (-0.34; 0.64) | 0.09  (-0.30; 0.47) | 0.13  (-0.49; 0.76) | **nEX** |
| **Health-related quality of life - Mental component score** | | | | | | |
| **In-person HIIT** | 0.08  (-0.16; 0.31) | . | . | . | . | 0.00  (-0.53; 0.53) |
| 0.00  (-0.22; 0.22) | **In-person MIT** | . | . | 0.48  (-0.05; 1.01) | . | **0.57**  **0.26; 0.89)** |
| 0.36  (-0.21; 0.93) | 0.36  (-0.18; 0.90) | **In-person MIT + R** | -0.16  (-0.91; 0.59) | 0.34  (-0.48; 1.16) | -0.07  (-0.61; 0.47) | 0.17  (-0.68; 1.03) |
| 0.44  (-0.07; 0.94) | 0.44  (-0.04; 0.91) | 0.08  (-0.44; 0.59) | **Home HIIT** | 0.00  (-0.52; 0.52) | . | -0.25  (-0.92; 0.41) |
| **0.42**  **0.02; 0.82)** | **0.42**  **0.06; 0.79)** | 0.06  (-0.44; 0.56) | -0.01  (-0.41; 0.38) | **Home MIT** | . | 0.08  (-0.27; 0.42) |
| 0.28  (-0.32; 0.88) | 0.28  (-0.30; 0.86) | -0.08  (-0.54; 0.38) | -0.15  (-0.75; 0.44) | -0.14  (-0.70; 0.42) | **Home MIT + R** | 0.12  (-0.56; 0.81) |
| **0.40**  **0.08; 0.71)** | **0.40**  **0.13; 0.67)** | 0.04  (-0.45; 0.52) | -0.04  (-0.45; 0.37) | -0.03  (-0.33; 0.28) | 0.11  (-0.40; 0.63) | **nEX** |
| Upper right triangle presents the pooled mean differences from direct comparisons and the lower left triangle pooled mean differences from the network meta-analysis. Columns are relative to the rows. Positive differences indicate an improvement in quality of life. | | | | | | |

**Supplemental Table 3.** League table with pooled SMD for in-person and home-based exercise on health-related quality of life for different durations of the interventions (≤12 weeks or >12 weeks).

| **Health-related quality of life - Total score** | | | | |
| --- | --- | --- | --- | --- |
| **In-person >12 weeks** | -0.09  (-1.00; 0.81) | 0.02  (-0.70; 0.75) | . | **0.76**  **(0.21; 1.31)** |
| 0.02 (-0.43; 0.48) | **In-person ≤12 weeks** | -0.09  (-1.00; 0.82) | 0.27  (-0.14; 0.67) | **0.55**  **(0.22; 0.87)** |
| 0.35 (-0.11; 0.81) | 0.32 (-0.08; 0.72) | **Home >12 weeks** | . | 0.02  (-0.38; 0.41) |
| 0.28 (-0.23; 0.79) | 0.26 (-0.08; 0.60) | -0.07 (-0.53; 0.39) | **Home ≤12 weeks** | 0.24  (-0.16; 0.63) |
| **0.53 (0.12; 0.95)** | **0.51 (0.23; 0.78)** | 0.19 (-0.15; 0.52) | 0.25 (-0.08; 0.59) | **nEX** |
| **Health-related quality of life - Physical component score** | | | | |
| **In-person >12 weeks** | . | 0.11 (-0.72; 0.95) | . | -0.13 (-1.10; 0.83) |
| -0.31 (-1.05; 0.44) | **In-person ≤12 weeks** | 0.07 (-0.85; 1.00) | 0.22 (-0.35; 0.79) | **0.40 (0.00; 0.81)** |
| -0.04 (-0.70; 0.61) | 0.27 (-0.24; 0.77) | **Home >12 weeks** | . | 0.11 (-0.40; 0.62) |
| -0.21 (-0.99; 0.58) | 0.10 (-0.35; 0.55) | -0.16 (-0.74; 0.41) | **Home ≤12 weeks** | 0.30 (-0.15; 0.75) |
| 0.07 (-0.61; 0.75) | **0.38 (0.02; 0.74)** | 0.11 (-0.31; 0.54) | 0.28 (-0.14; 0.69) | **nEX** |
| **Health-related quality of life - Mental component score** | | | | |
| **In-person >12 weeks** | . | -0.07 (-0.78; 0.63) | . | 1.19 (0.44; 1.94) |
| 0.23 (-0.39; 0.84) | **In-person ≤12 weeks** | -0.16 (-1.04; 0.72) | 0.45 (-0.02; 0.93) | **0.40 (0.07; 0.74)** |
| 0.42 (-0.12; 0.96) | 0.19 (-0.25; 0.64) | **Home >12 weeks** | . | -0.08 (-0.53; 0.36) |
| 0.50 (-0.15; 1.14) | 0.27 (-0.11; 0.65) | 0.07 (-0.42; 0.57) | **Home ≤12 weeks** | 0.24 (-0.15; 0.62) |
| **0.63 (0.08; 1.18)** | **0.40 (0.10; 0.70)** | 0.21 (-0.17; 0.58) | 0.13 (-0.21; 0.48) | **nEX** |

The analyses for intervention duration included 19 studies for overall score of HR-QoL and 14 for the PCS and MCS.

**Supplemental Table 4.** League table with pooled SMD for in-person and home-based exercise on health-related quality of life for different total intervention volumes (≤24 hours or >24 hours).

| **Health-related quality of life - Total score** | | | | | | | | |
| --- | --- | --- | --- | --- | --- | --- | --- | --- |
| **In-person >24 hrs** | -0.01  (-0.56; 0.54) | | | 0.23  (-0.33; 0.79) | . | | | **0.65**  **(0.17; 1.13)** |
| -0.00  (-0.38; 0.38) | **In-person ≤24 hrs** | | | . | 0.31  (-0.25; 0.87) | | | **0.58**  **(0.22; 0.94)** |
| **0.46**  **(0.06; 0.86)** | **0.46**  **(0.04; 0.89)** | | | **Home >24 hrs** | . | | | -0.03  (-0.38; 0.33) |
| 0.36  (-0.17; 0.89) | 0.37  (-0.08; 0.82) | | | -0.10  (-0.63; 0.44) | **Home ≤24 hrs** | | | 0.16  (-0.34; 0.66) |
| **0.54**  **(0.19; 0.88)** | **0.54**  **(0.23; 0.85)** | | | 0.08  (-0.25; 0.40) | 0.17  (-0.26; 0.61) | | | **nEX** |
| **Health-related quality of life - Physical component score** | | | | | | | | |
| **In-person >24 hrs** | 0.02 (-0.79; 0.83) | | | 0.37 (-0.69; 1.43) | . | | | 0.46 (-0.29; 1.20) |
| 0.11 (-0.47; 0.68) | **In-person ≤24 hrs** | | | . | 0.16 (-0.50; 0.82) | | | 0.38 (-0.09; 0.85) |
| 0.40 (-0.26; 1.05) | 0.29 (-0.30; 0.89) | | | **Home >24 hrs** | . | | | 0.02 (-0.43; 0.48) |
| 0.24 (-0.48; 0.95) | 0.13 (-0.41; 0.67) | | | -0.16 (-0.84; 0.51) | **Home ≤24 hrs** | | | 0.25 (-0.33; 0.83) |
| 0.45 (-0.10; 1.01) | 0.35 (-0.06; 0.76) | | | 0.05 (-0.39; 0.50) | 0.22 (-0.30; 0.73) | | | **nEX** |
| **Health-related quality of life - Mental component score** | | | | | | | | |
| **In-person >24 hrs** | | 0.06 (-0.46; 0.58) | 0.34 (-0.46; 1.15) | | | . | **0.51 (0.01; 1.01)** | |
| 0.10 (-0.28; 0.47) | | **In-person ≤24 hrs** | . | | | **0.49 (0.07; 0.91)** | **0.36 (0.08; 0.65)** | |
| **0.54 (0.09; 0.99)** | | **0.44 (0.05; 0.84)** | **Home >24 hrs** | | | . | -0.10 (-0.41; 0.22) | |
| 0.46 (-0.01; 0.92) | | 0.36 (0.02; 0.70) | -0.08 (-0.53; 0.36) | | | **Home ≤24 hrs** | 0.15 (-0.22; 0.52) | |
| **0.47 (0.10; 0.84)** | | **0.37 (0.12; 0.63)** | -0.07 (-0.38; 0.24) | | | 0.01 (-0.32; 0.34) | **nEX** | |

The analyses for intervention volume included 16 studies for overall score of HR-QoL and 11 for the PCS and MCS.

**
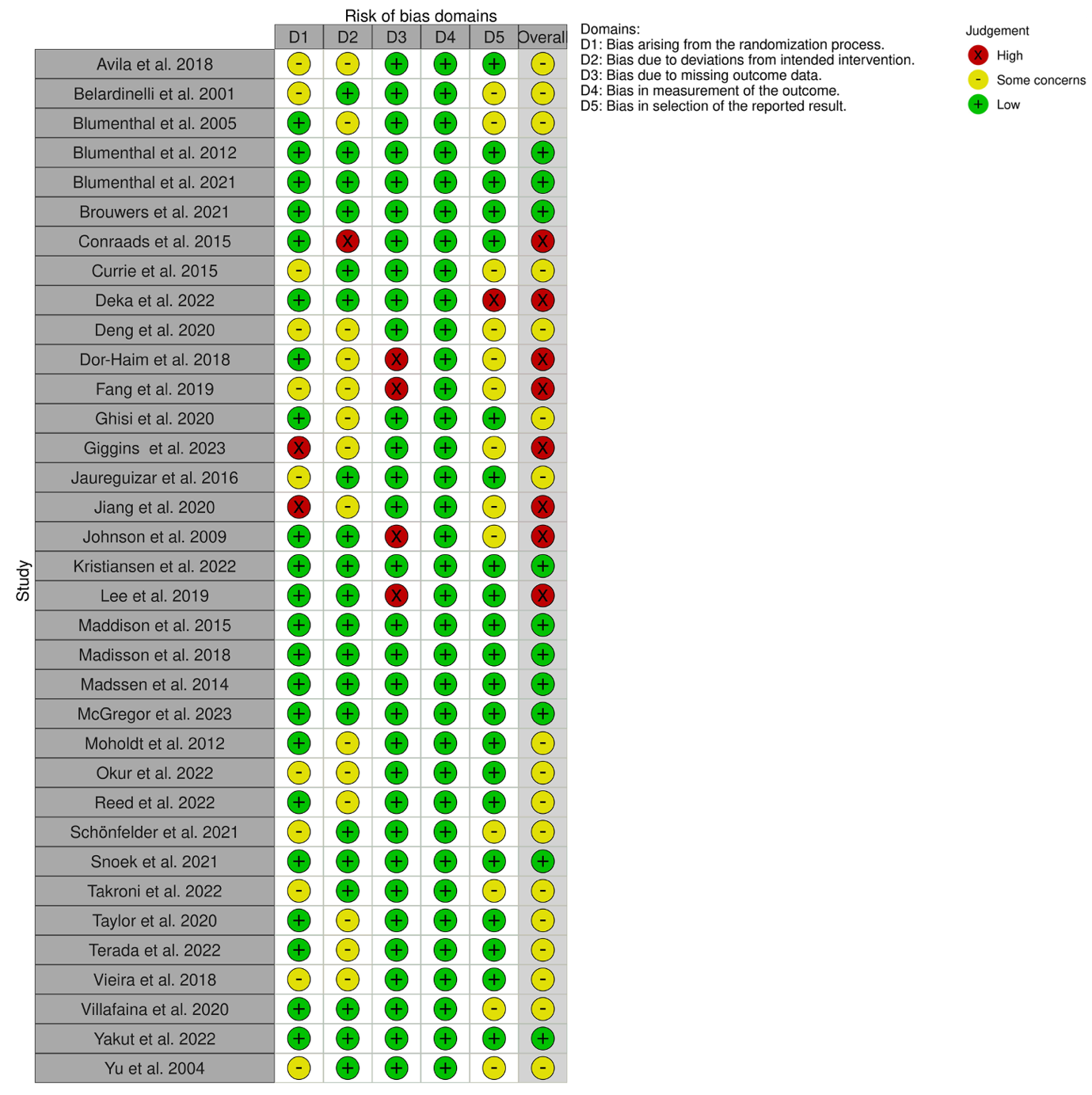
**

**Supplemental** **Figure 1**. Risk of bias of the included study using the ROB2.

**
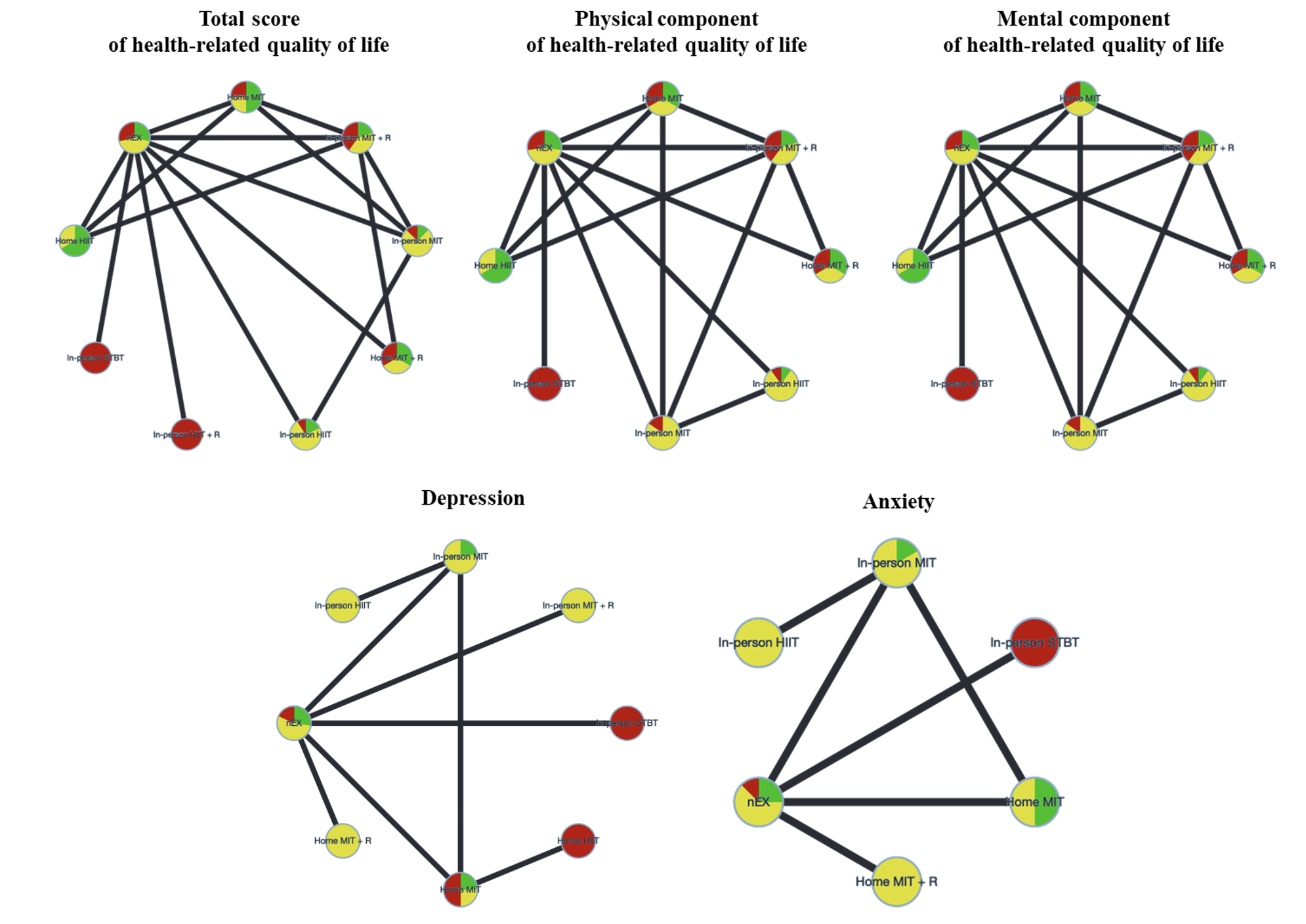
**

**Supplemental figure 2**. Network of eligible comparisons for different brain-related outcomes showing the Confidence in Network Meta-Analysis (CINeMA) to evaluate the certainty of the evidence. Green is low risk bias, yellow is some concerns and red is high risk of bias based on ROB2 tool.

**
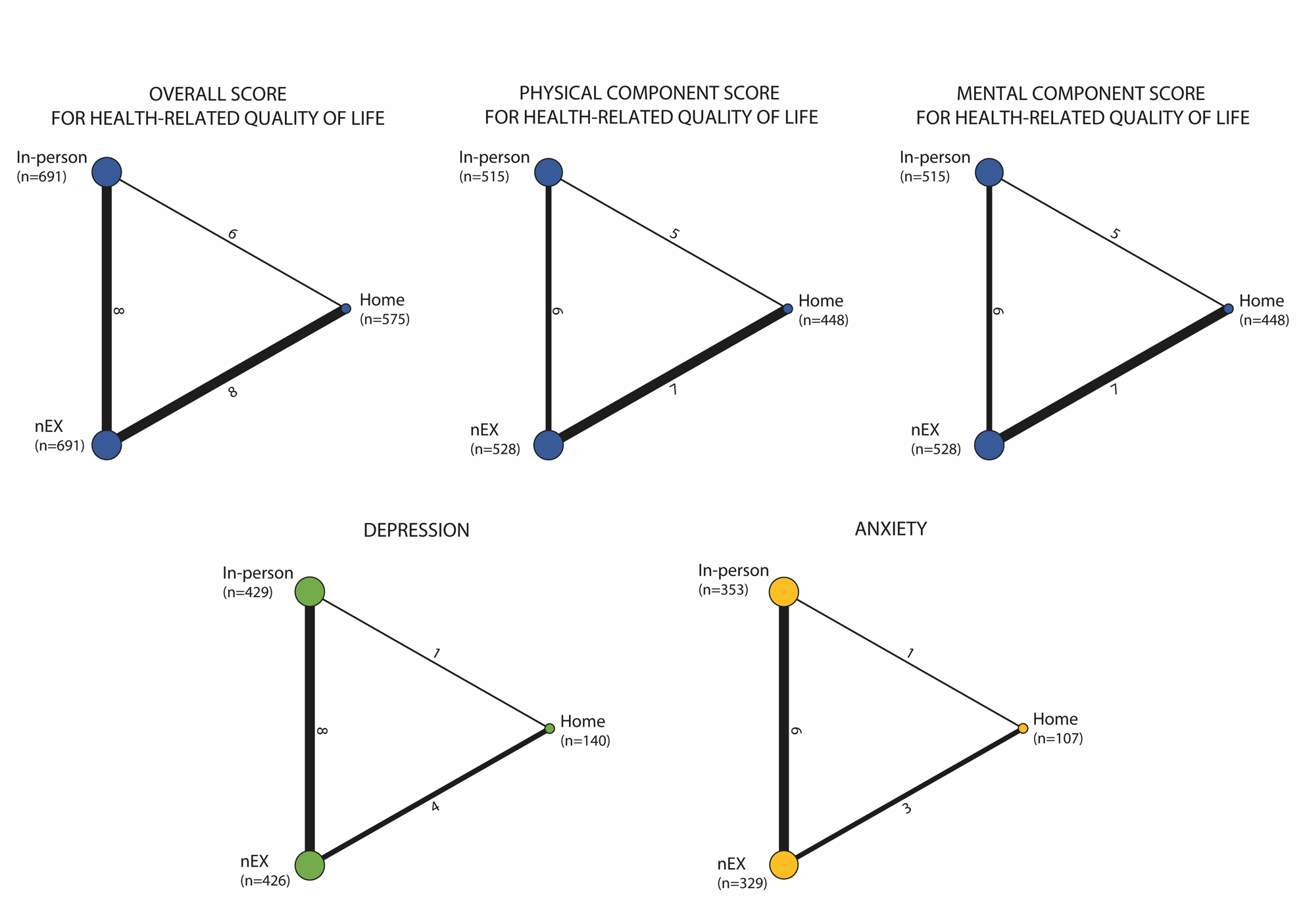
**

**Supplemental figure 3**. Network of eligible comparisons for different brain-related outcomes.
The width of the lines is proportional to the number of trials comparing each pair of treatments. The nodes represented the different interventions, node size was proportional to the number of participants, and the connecting lines showed the direct comparisons in which the thickness indicates frequency of the comparison. For analyses, the following number of studies were included: (i) total score HR-QoL in 18 studies, (ii) PCS of HR-QoL in 14 studies, (iii) MCS in 14 studies, (iv) depression in 11 studies, and (v) anxiety in 8 studies.


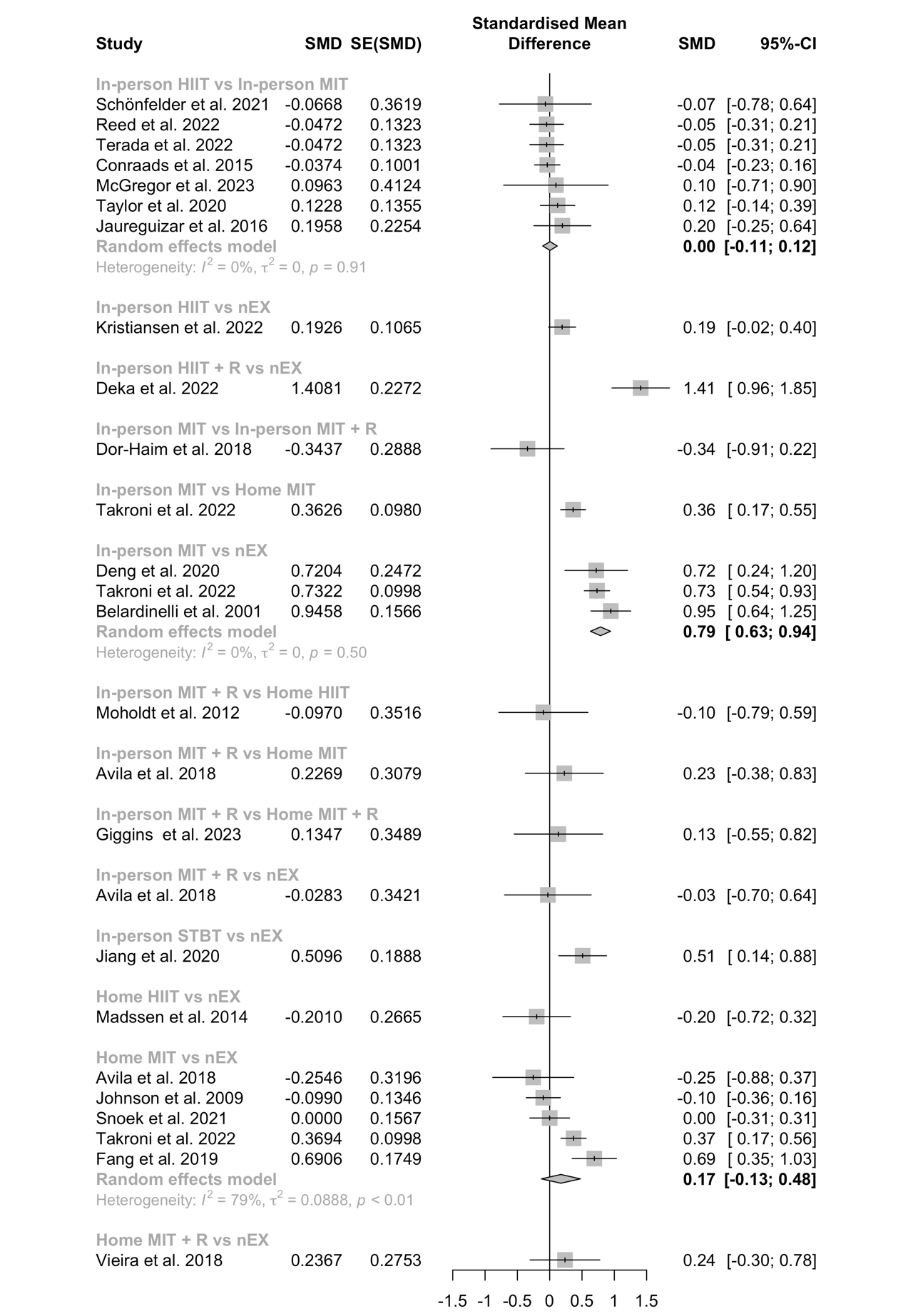
**Supplemental Figure 4.** Pair-wise comparison of studies including the total score of health-related quality of life. Note: The pooled effect size was estimated (diamonds in the forest plot) only when two or more estimates for a same comparison were available.


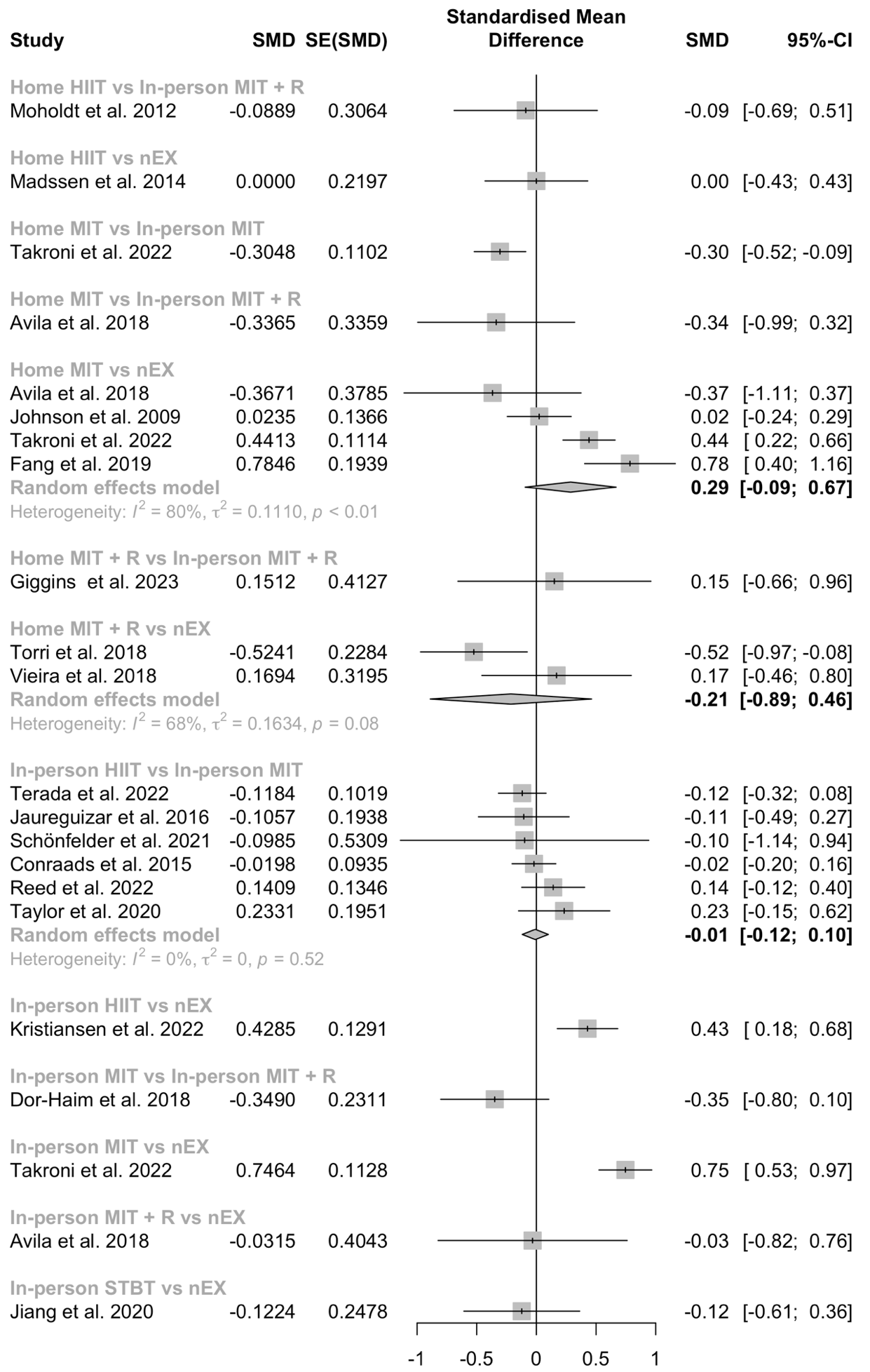


**Supplemental Figure 5.** Pair-wise comparison of studies including the physical component score of health-related quality of life.

**
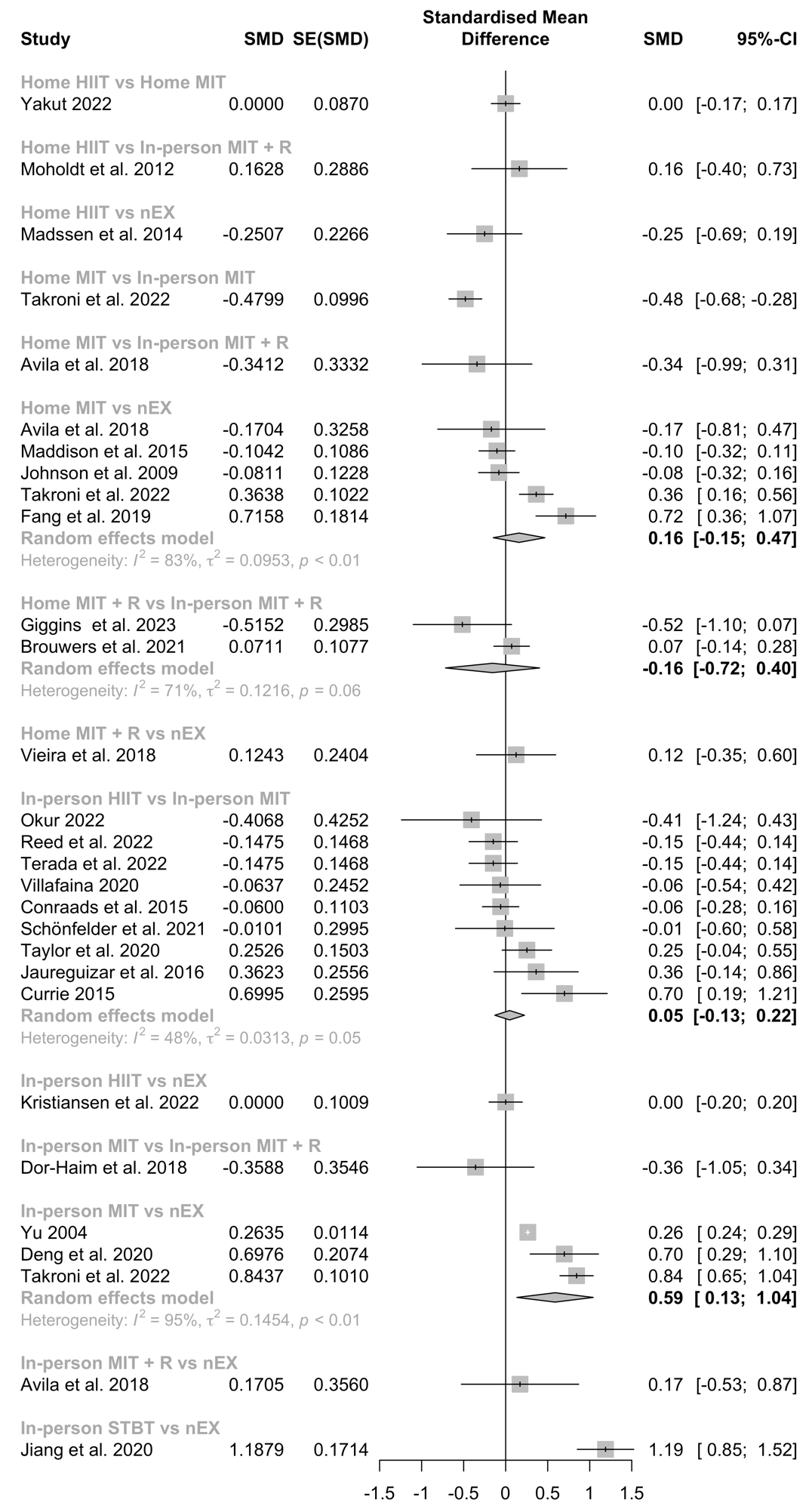
**

**Supplemental Figure 6.** Pair-wise comparison of studies including the mental component score of health-related quality of life.


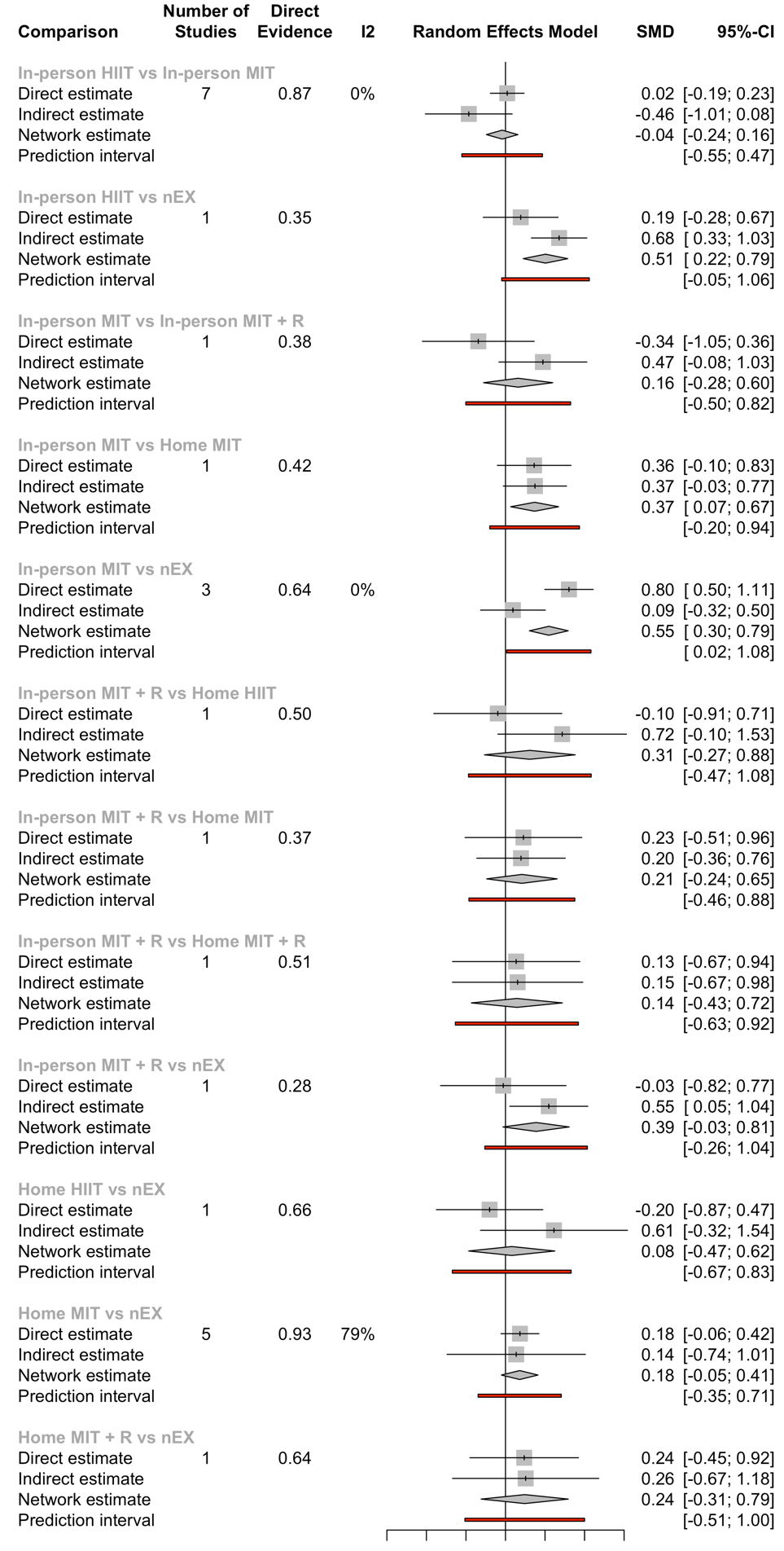


**Supplemental Figure 7.** Direct and indirect effect estimates for studies including the total score of health-related quality of life. P value for global inconsistency is 0.71.

**
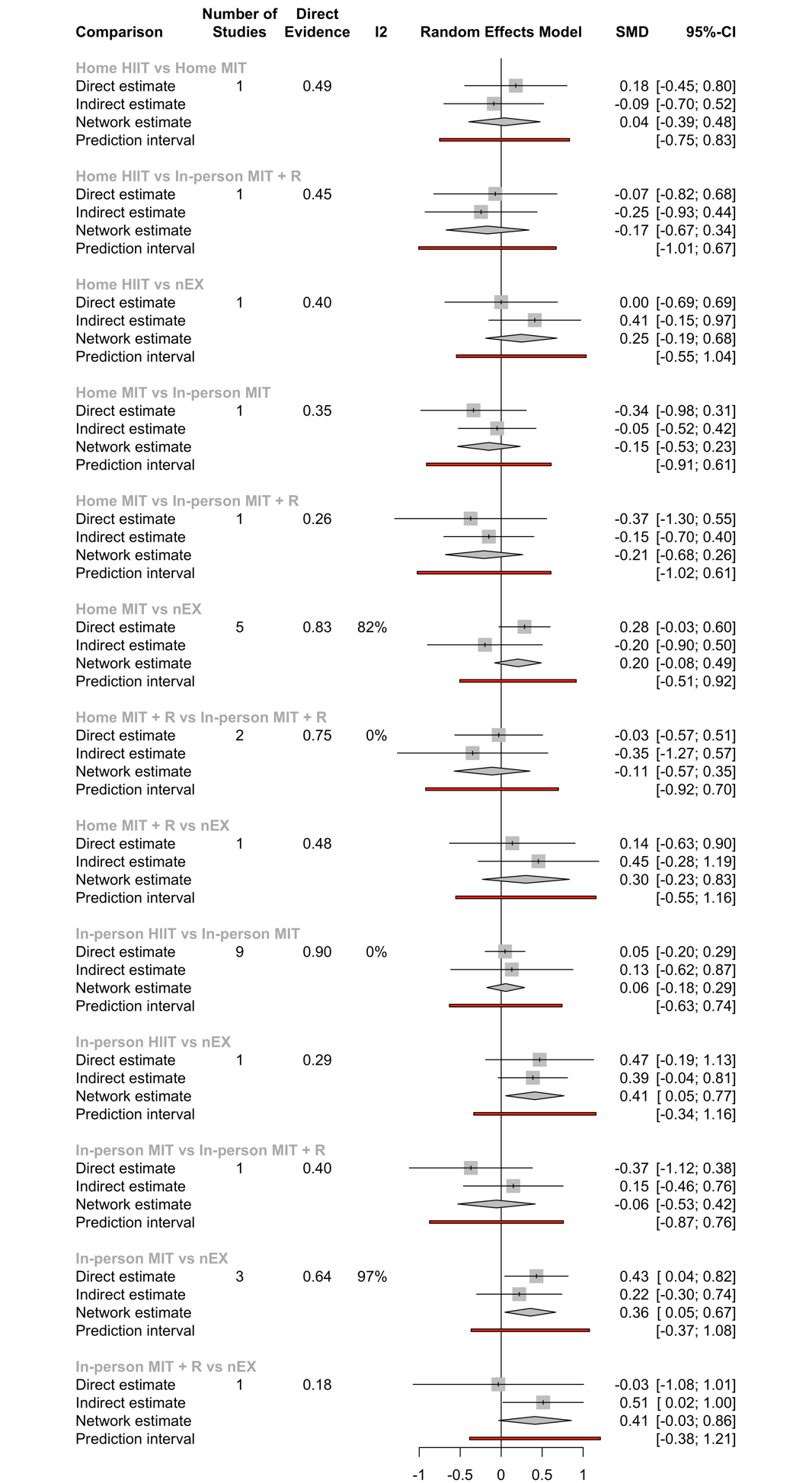
**

**Supplemental Figure 8.** Direct and indirect effect estimates for studies including the physical component score of health-related quality of life. P value for global inconsistency is 0.18.


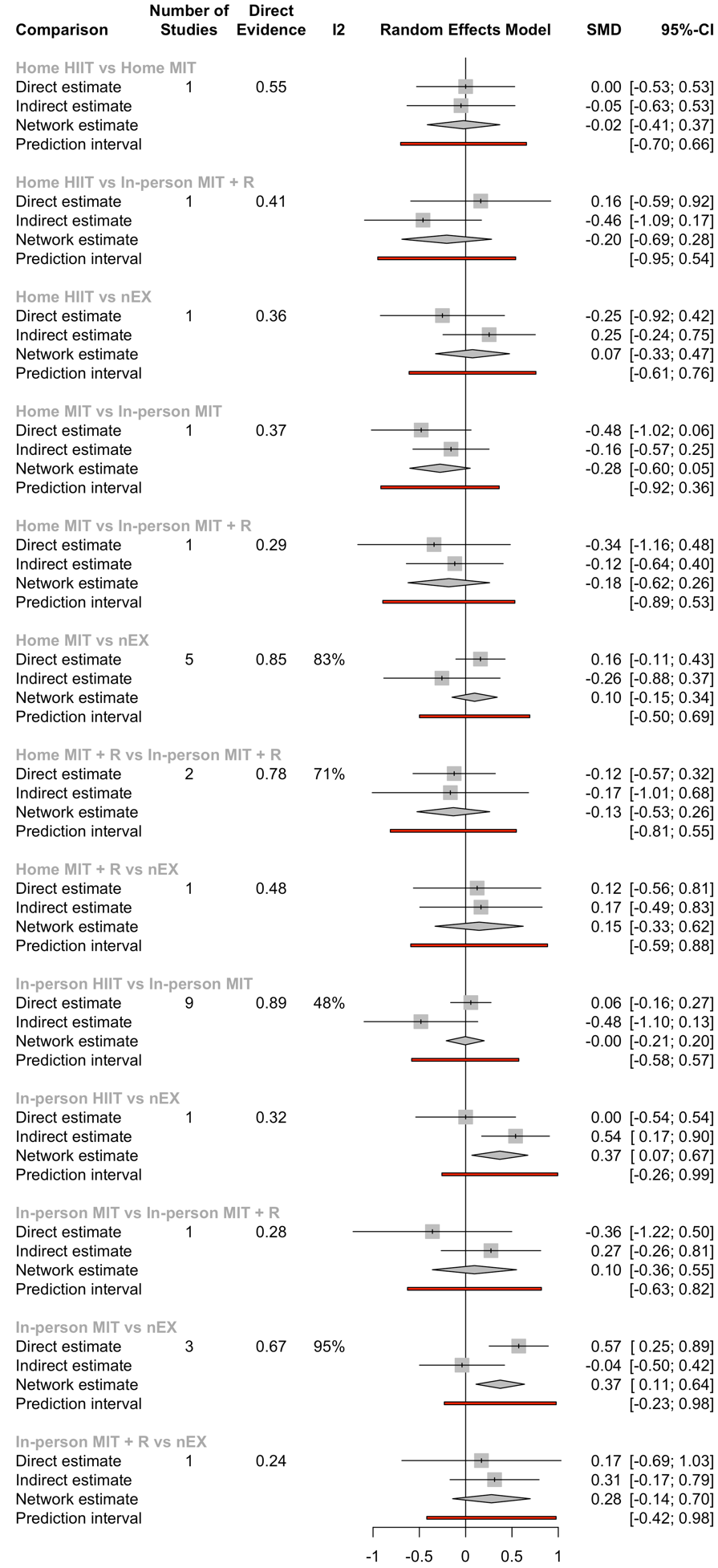


**Supplemental Figure 9.** Direct and indirect effect estimates for studies including the mental component score of health-related quality of life. P value for global inconsistency is 0.40.

| **Total score of health-related quality of life** | |
| --- | --- |
| **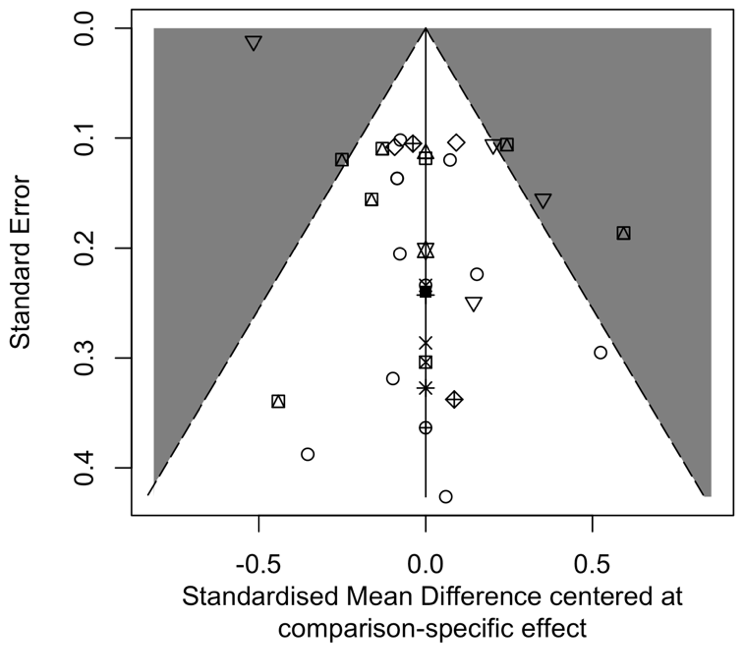** | **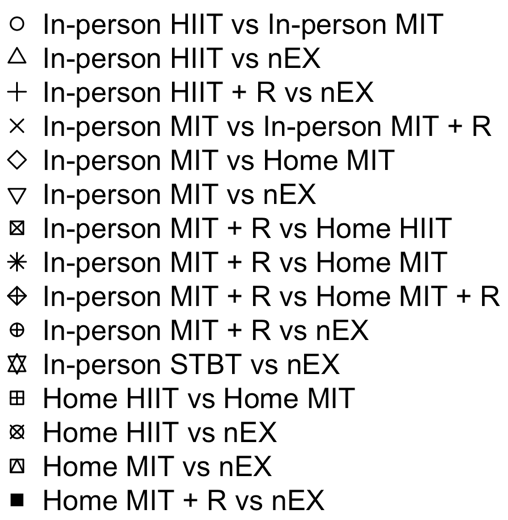** |
| **Physical component score of health-related quality of life** | |
| **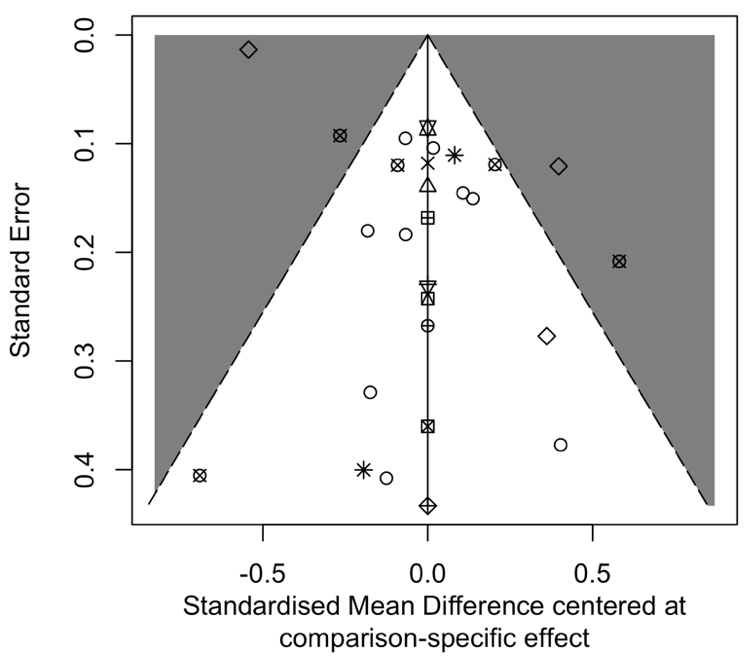** | **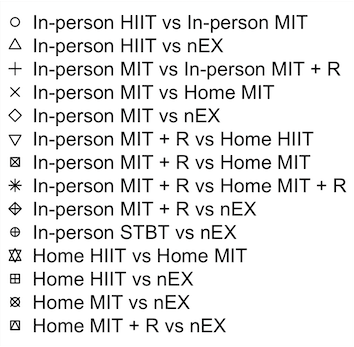** |
| **Mental component score of health-related quality of life** | |
| **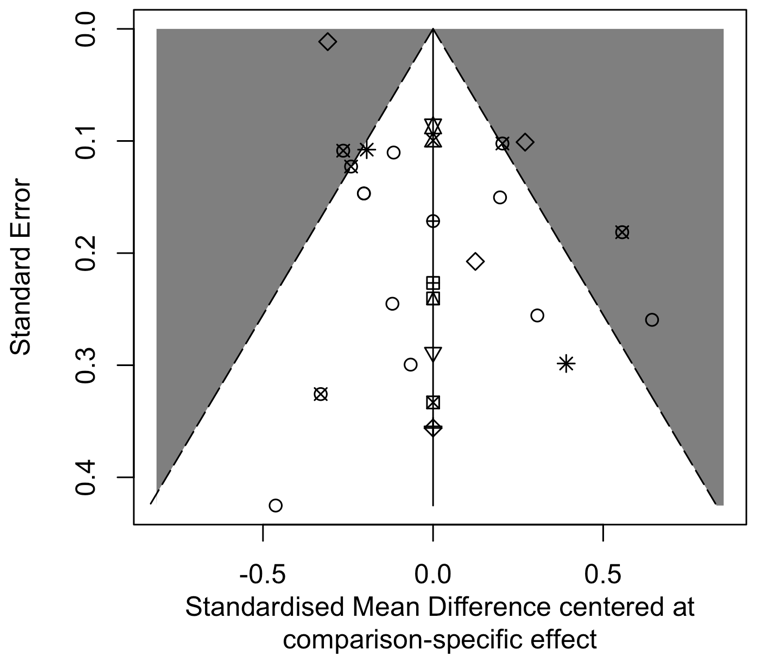** | **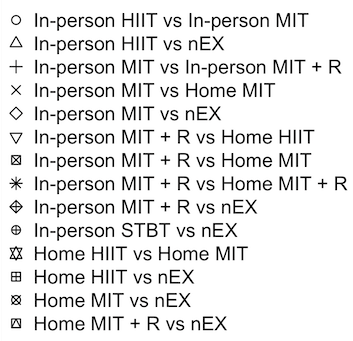** |
| **Supplemental Figure 10.** Funnel plots for all HR-QoL outcomes. | |
