# Supplementary material for: Exercise type and settings, quality of life, and mental health in coronary artery disease: a network meta-analysis
Source: Eur Heart J. 2025 Jan 15;46(23):2186–201. doi: 10.1093/eurheartj/ehae870 (PMC12167663; doi:10.1093/eurheartj/ehae870)
Supplement: ehae870_Supplementary_Data [file ehae870_supplementary_data.zip › Supplemental File 3. Tables figures_part 2_R1_clean.docx]

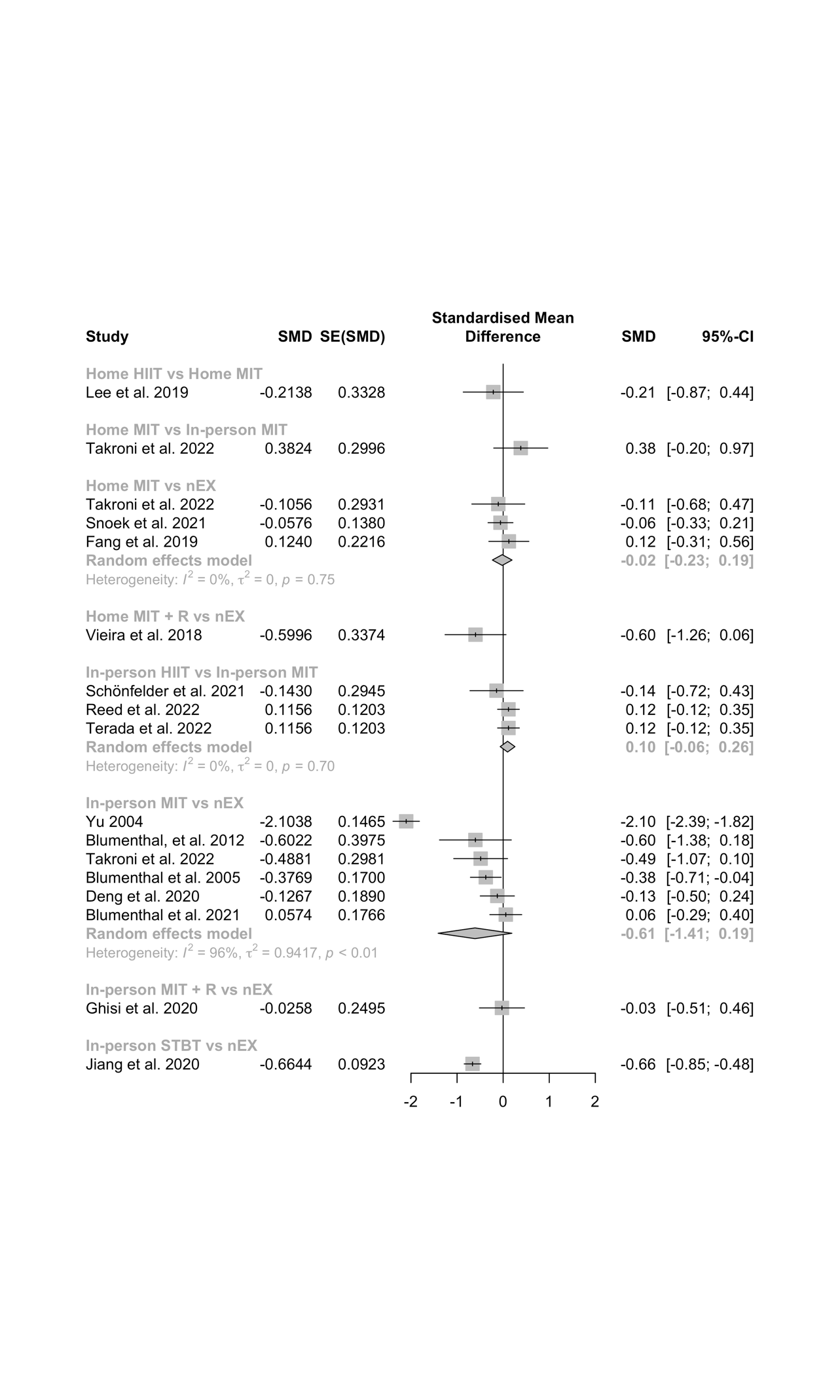


**Supplemental Figure 11.** Pair-wise comparison of studies including depression.


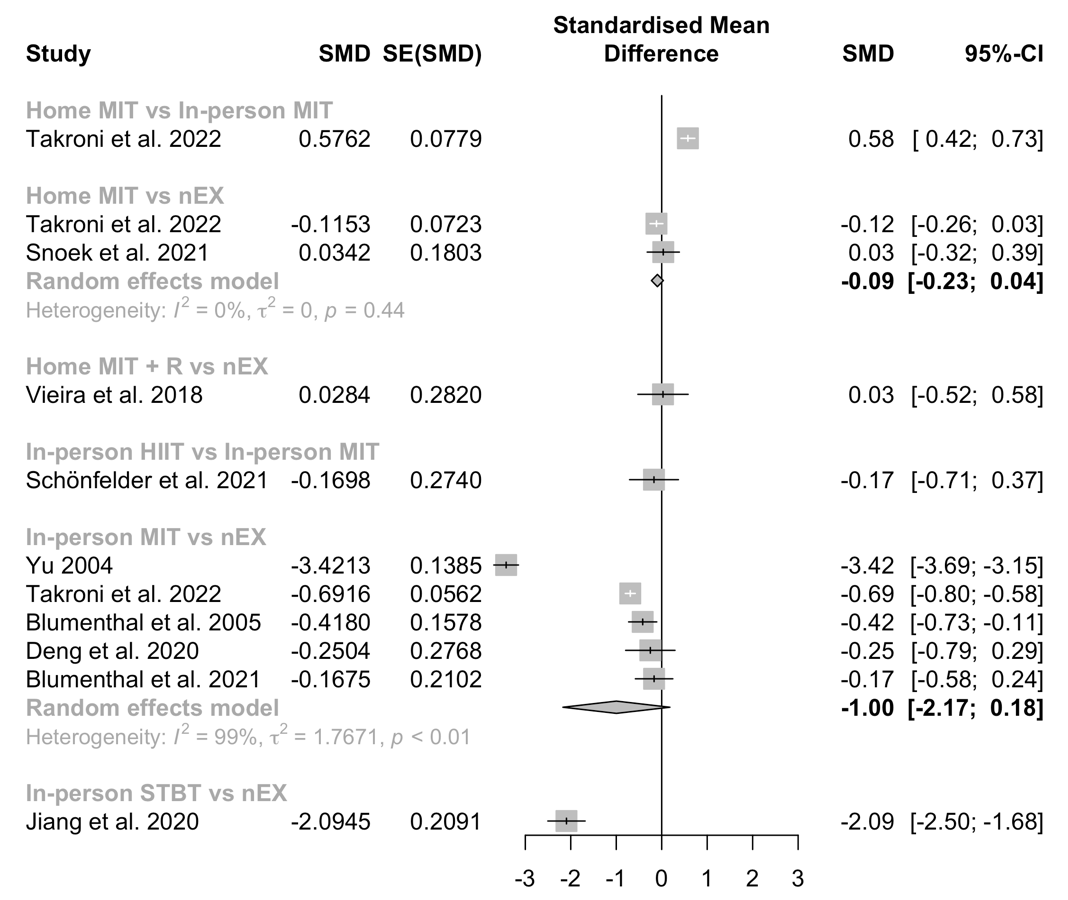


**Supplemental Figure 12.** Pair-wise comparison of studies including anxiety.


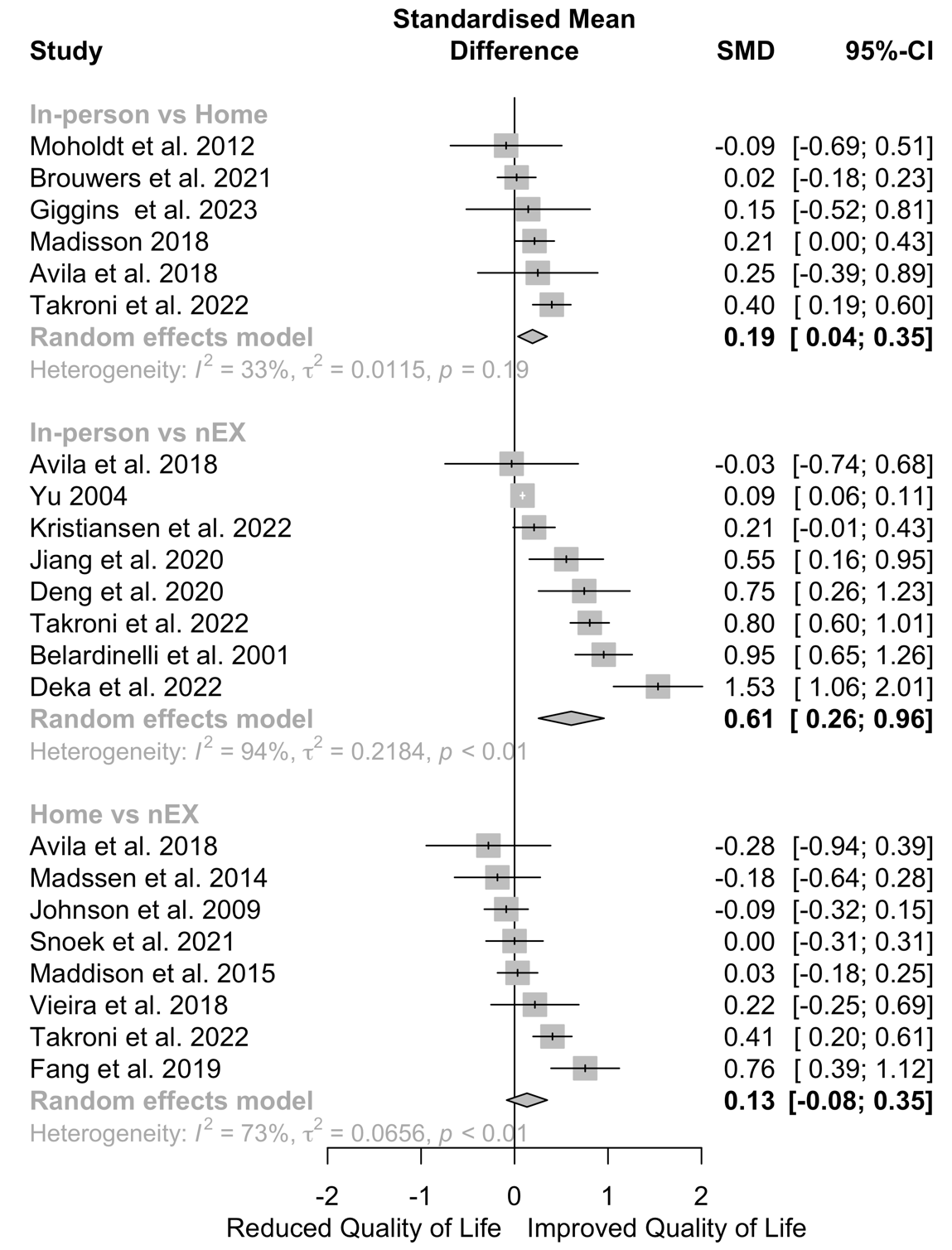


**Supplemental Figure 13.** Pair-wise comparison of studies including in-person, home, or no exercise and the total score of health-related quality of life.

**
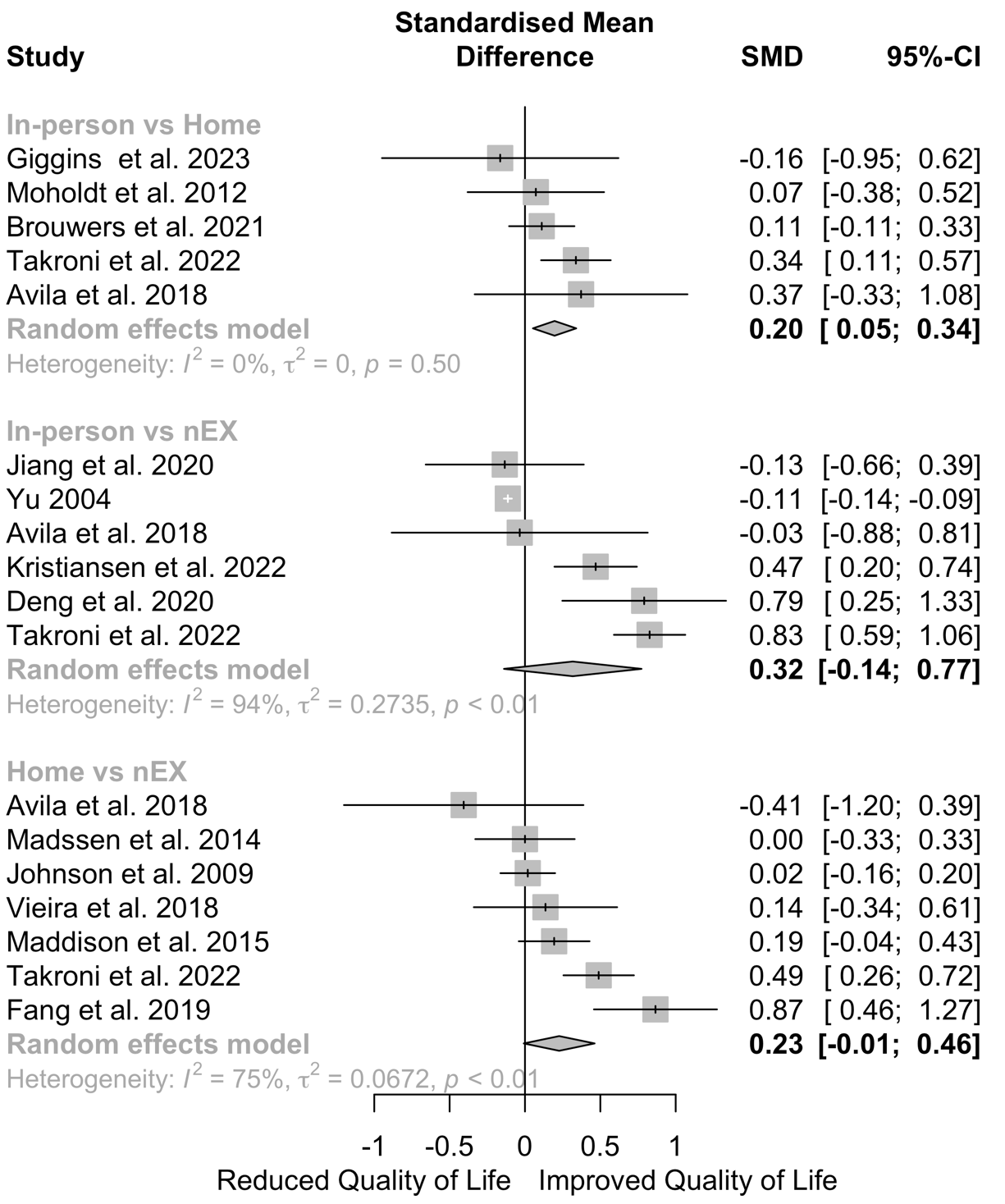
**

**Supplemental Figure 14.** Pair-wise comparison of studies including in-person, home, or no exercise and the physical component score of health-related quality of life.

**
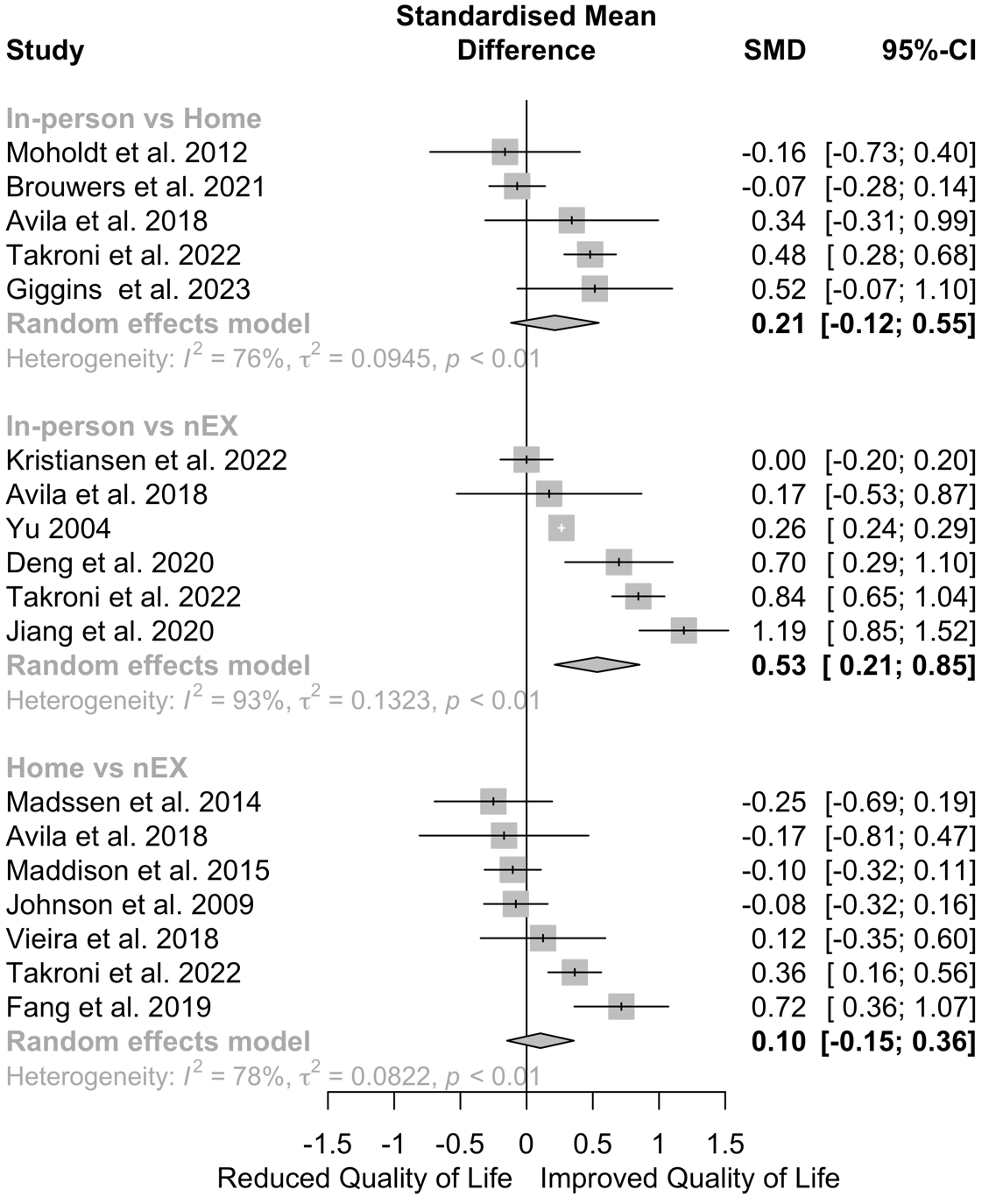
**

**Supplemental Figure 15.** Pair-wise comparison of studies including in-person, home, or no exercise and the mental component score of health-related quality of life.

**
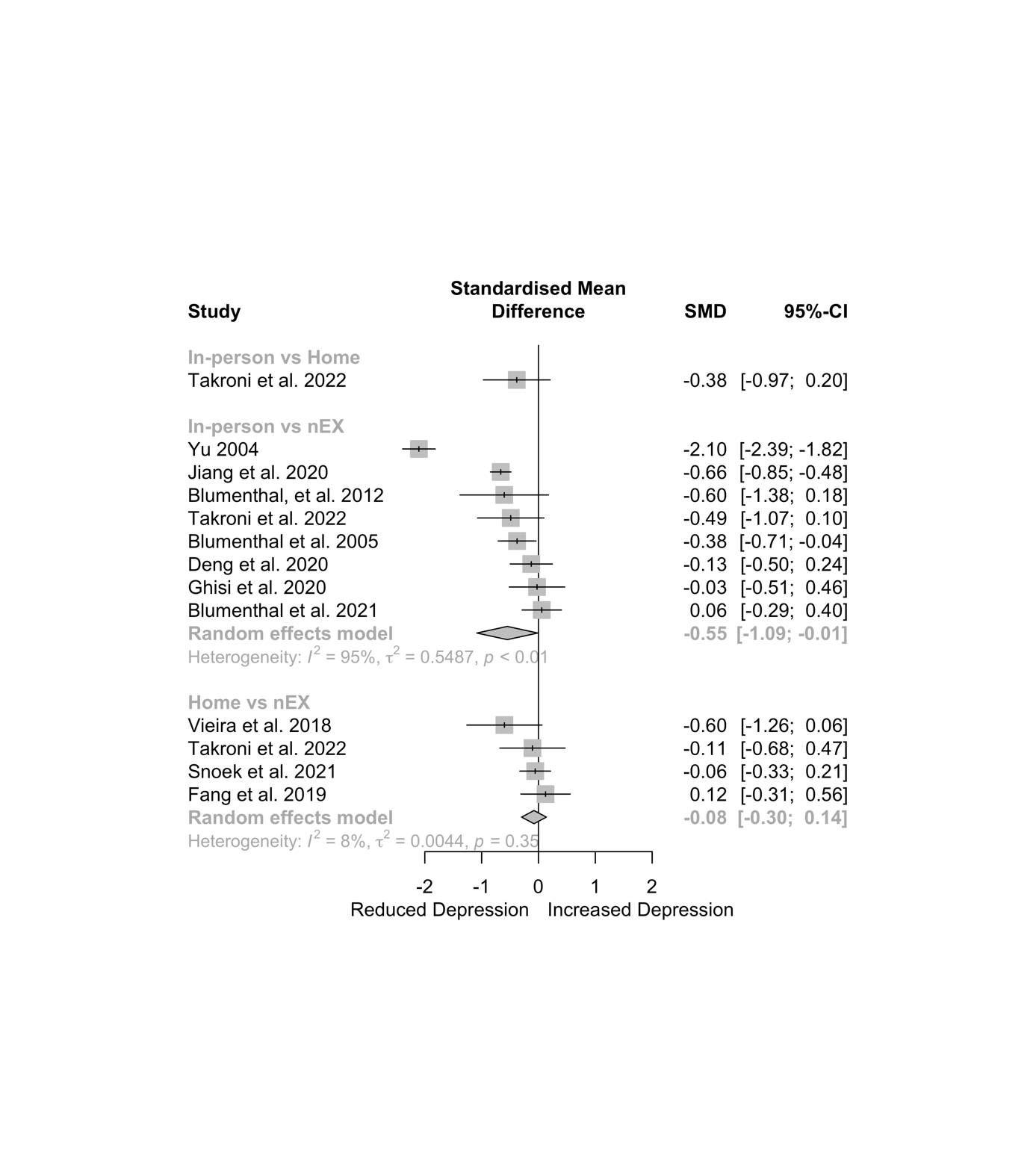
**

**Supplemental Figure 16.** Pair-wise comparison of studies including in-person, home, or no exercise and depression.


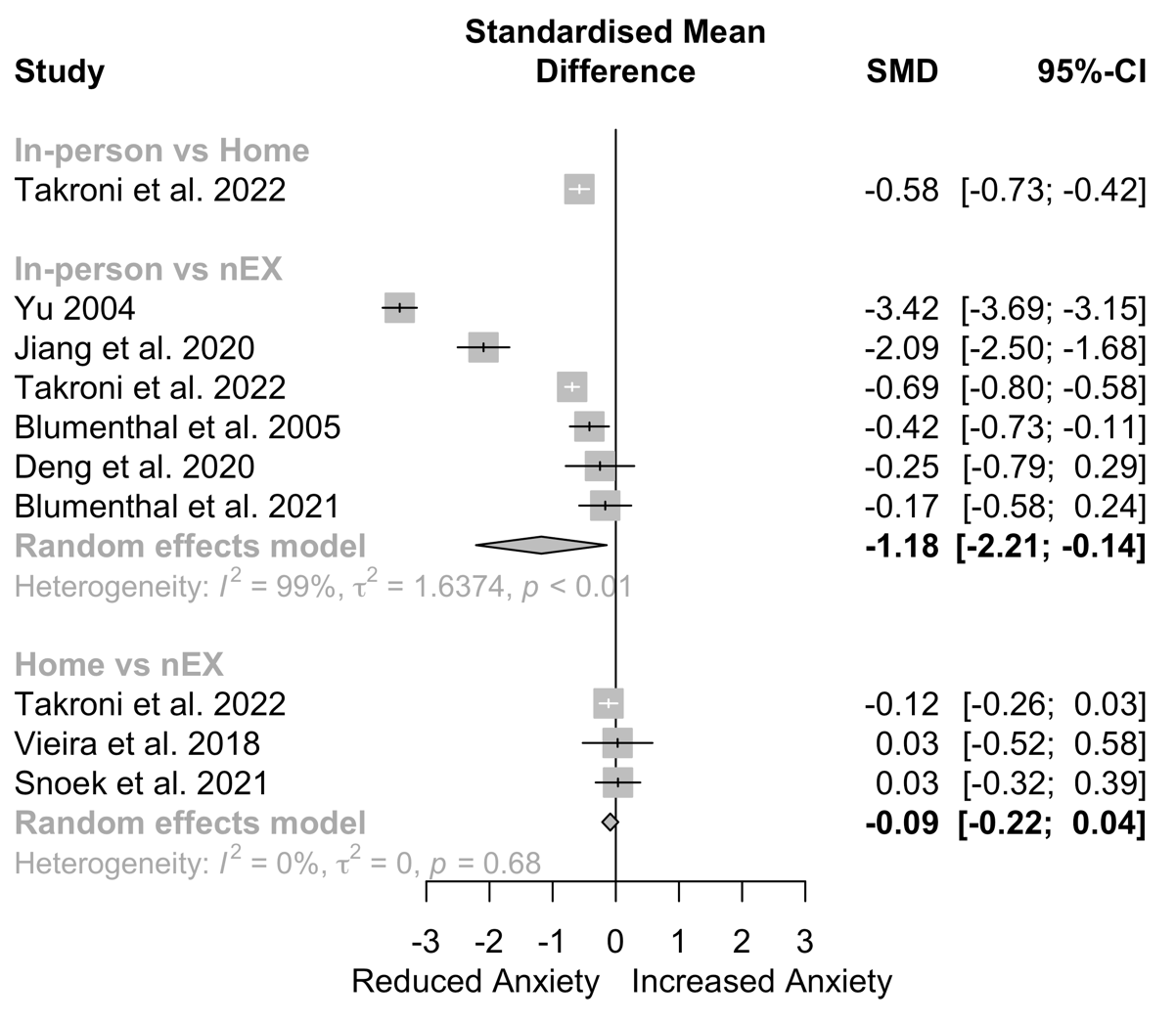


**Supplemental Figure 17.** Pair-wise comparison of studies including in-person, home, or no exercise and anxiety.


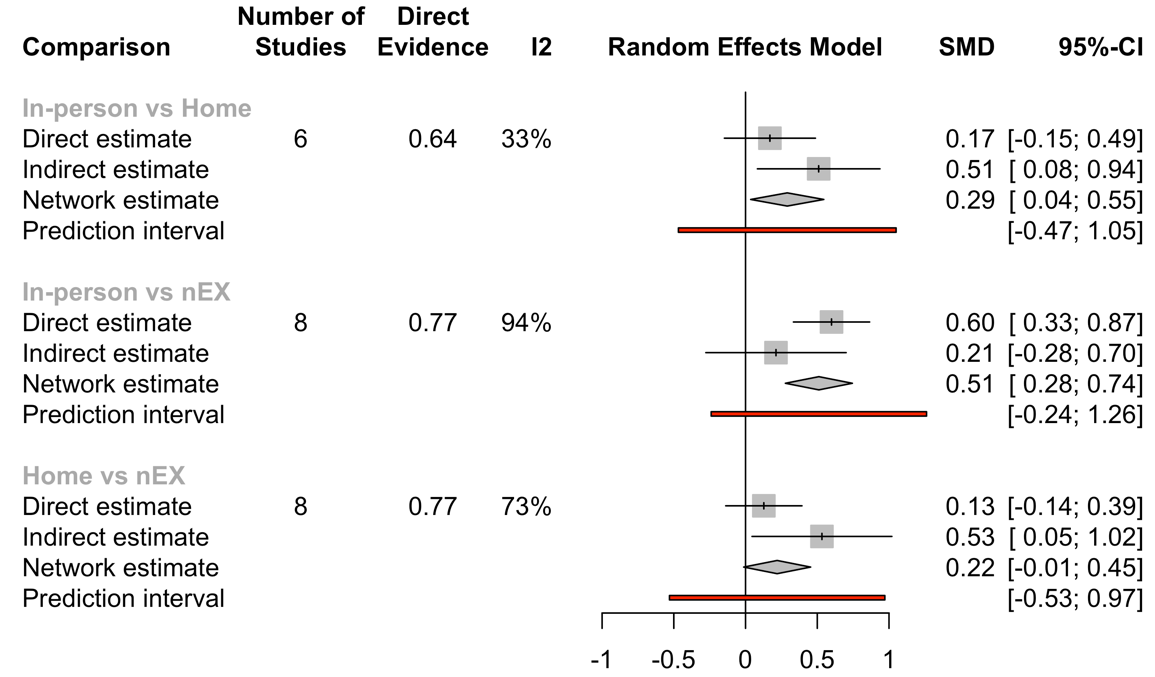


**Supplemental Figure 18.** Direct and indirect effect estimates for studies including the total score of health-related quality of life. P value for global inconsistency is 0.58.


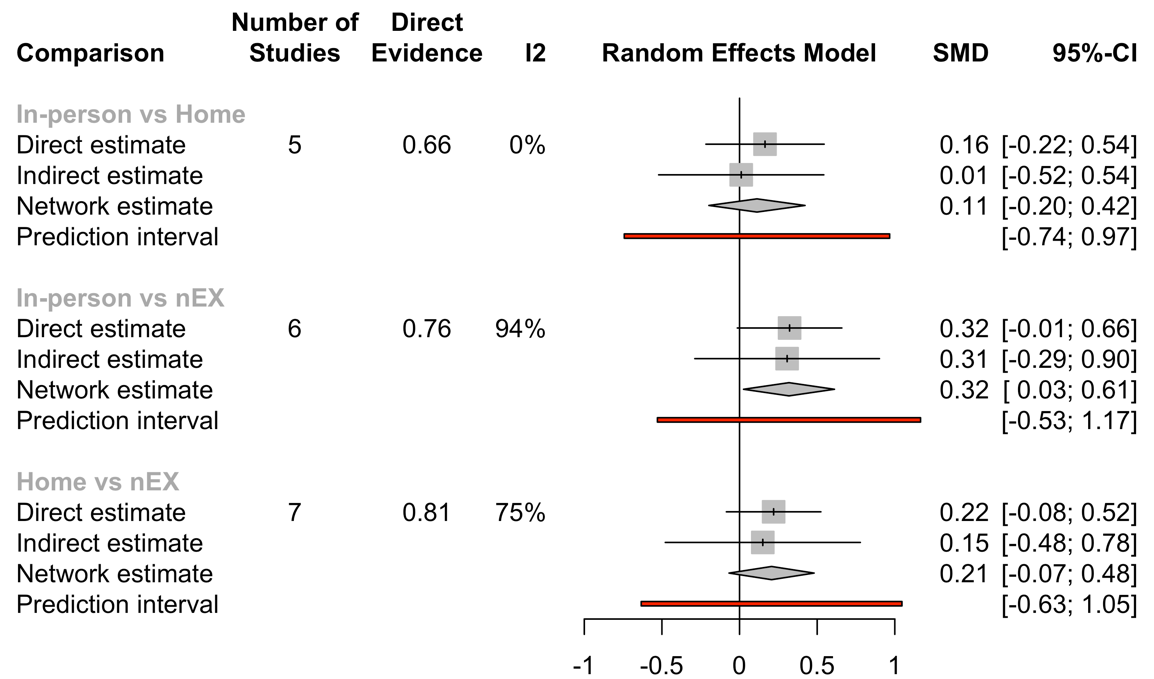


**Supplemental Figure 19.** Direct and indirect effect estimates for studies including the physical component score of health-related quality of life. P value for global inconsistency is 0.63.


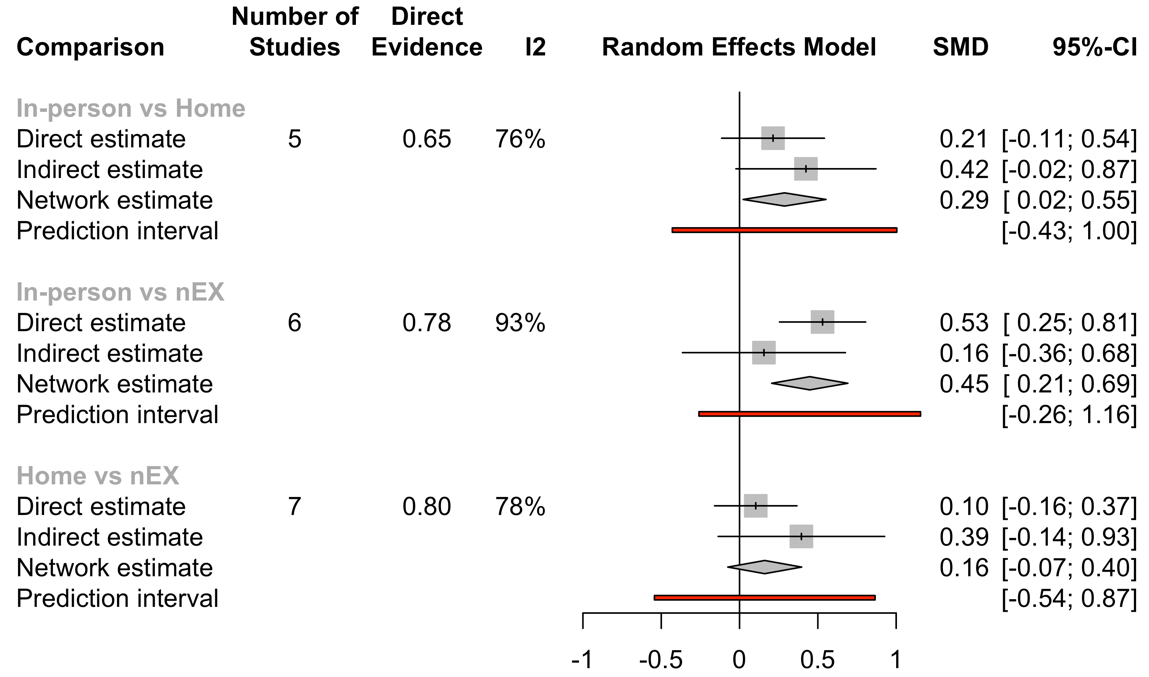


**Supplemental Figure 20.** Direct and indirect effect estimates for studies including the mental component score of health-related quality of life. P value for global inconsistency is 0.64.

**
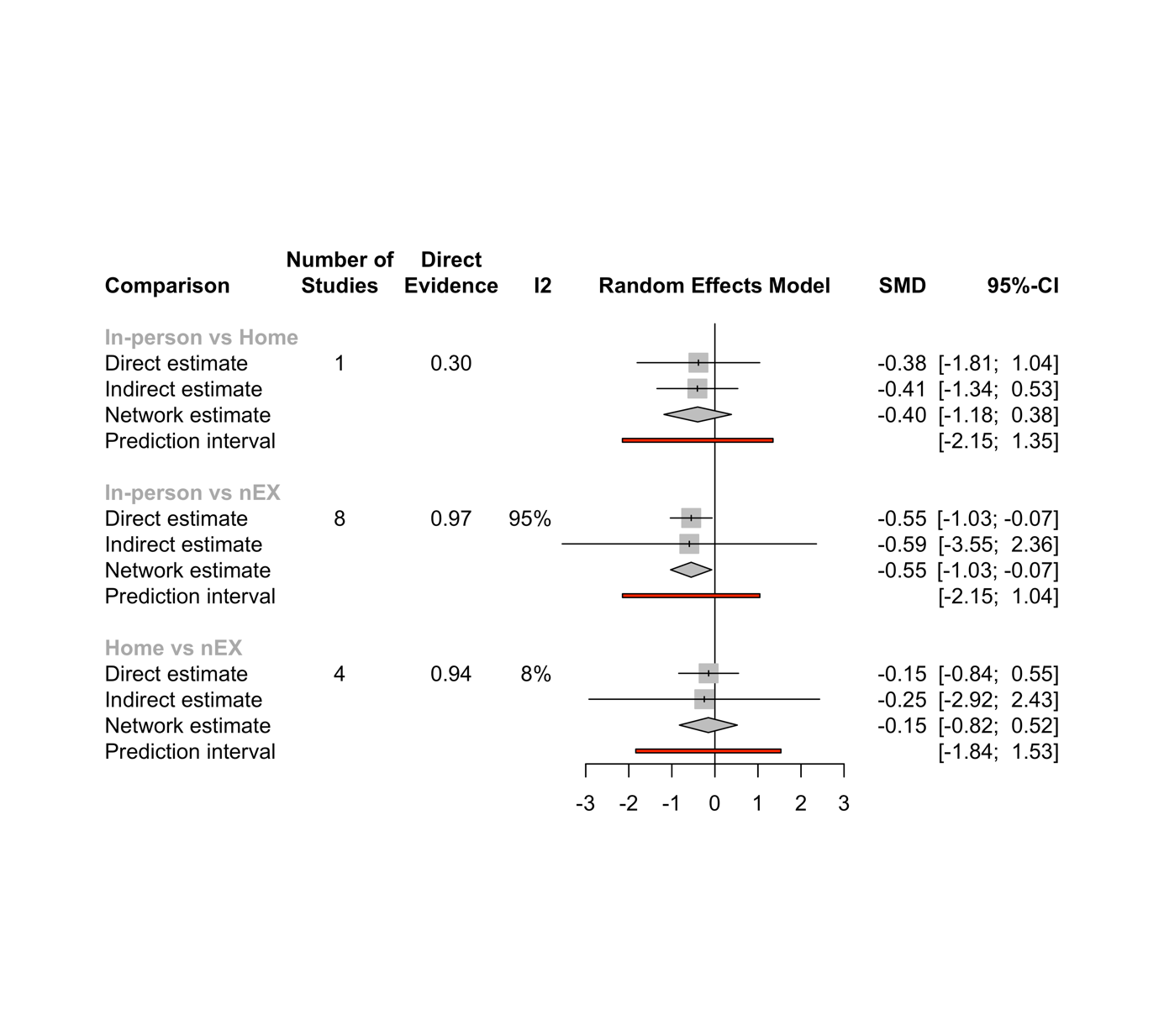
**

**Supplemental Figure 21.** Direct and indirect effect estimates for studies including depression. P value for global inconsistency is 0.996.


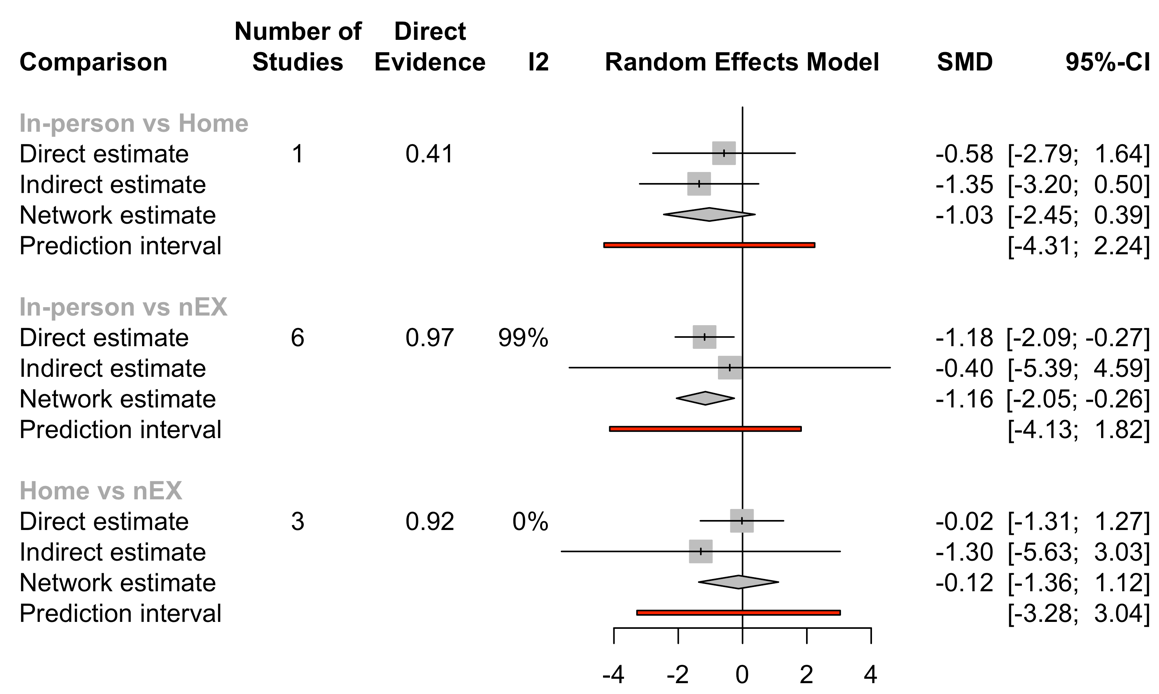


**Supplemental Figure 22.** Direct and indirect effect estimates for studies including anxiety. P value for global inconsistency is 0.92.
