# Supplementary material for: Exercise type and settings, quality of life, and mental health in coronary artery disease: a network meta-analysis
Source: Eur Heart J. 2025 Jan 15;46(23):2186–201. doi: 10.1093/eurheartj/ehae870 (PMC12167663; doi:10.1093/eurheartj/ehae870)
Supplement: ehae870_Supplementary_Data [file ehae870_supplementary_data.zip › Supplemental_File_2_Extended_Methods_R2.docx]

**Supplemental File 2. Extended Methods**

**Data extraction.**

The following information was extracted based on the PICOS strategy:

*Population and study characteristics:* author, publication year, country, study design, mental status, sample size (total and from each intervention arm) at baseline and follow up, percentage of women and age at baseline.

*Intervention.* Exercise type (using a pre-established coding guide, **Table 1**), setting (i.e., in-person or home-based), duration of the intervention, frequency (sessions/week), duration of the sessions and exercise intensity (i.e., moderate or high). Furthermore, the time between the CAD diagnosis and intervention was extracted.

*Comparison.* Description of the comparator (control) group.

*Outcomes*. Information about the brain health dimensions including health-related QoL (i.e., overall, physical and mental components), mental health (i.e., anxiety, depression, well-being, stress), sleep quality, cognition (i.e., executive function and memory) or biomarkers of brain structure/function. Health-related QoL, depression and anxiety were included in the network meta-analysis. For the network meta-analysis, the mean change-from-baseline values, pre- and post-intervention values were extracted.

Exercise interventions types were divided in five categories based on previous studies (1):

**Data processing**

For the main analyses regarding health-related QoL, we included the overall and component scores (i.e., mental [MCS], and physical [PCS]). When the overall or component scores were not reported, the domain scores, or PCS and MCS were pooled following the guidelines of the corresponding questionnaires (2, 3). Some studies did not include a MCS but reported the social component score (SCS) and the emotional component score (ECS). For these studies, we estimated the MCS by combining the SCS and ECS using pooling (i.e. aggregating) with equal weights. In case of the SF-36, a total score is not included in the questionnaire guidelines (2) . However, since most other studies reported a total score, we estimated the total score by pooling the PCS and MCS using equal weights for each component.

Mean change-from-baseline values were standardised using the weighted pooled pre-intervention SD, as is recommended for patients-reported outcomes(4), and converted to Hedges’ g using the correction factor J(5) as most studies included a relatively low sample size. Several studies reported two instruments for the same construct or outcome. Therefore, we ranked the instruments based on the content of the instrument, overall use in the literature, and corresponding validation studies. For QoL, we used the following ranking: 1) SFs (8, 12 and 36), MacNew, Quality of Life after Myocardial Infarction, HeartQoL and Cardiovascular Prevention & Rehabilitation Medical Health Questionnaire, 2) MOS, 3) EQ-5D, DASI, EQ-5D-5L, and EQ-VAS. When two instruments were included or had a similar ranking, both instruments were pooled with equal weights. For anxiety and depression, all instruments were included. When studies included similar types of exercise within the intervention arms, the intervention arms were pooled using the sample size of the different arms.

**Statistical analyses**

All NMA were based on frequentist framework, used inverse variance weighting for each pairwise comparison (6) and included methods derived from graph theory (7) to estimate the exercise intervention effects. Furthermore, we used restricted maximum likelihood to estimate the between-study variance. In addition, 95% prediction interval (prI) were estimated.

The main NMA assumptions (i.e. transitivity and consistency) were considered. Transitivity was checked by comparing the distribution of potential effect modifiers (e.g. age, sex, time between diagnosis and exercise intervention) across intervention comparisons. The consistency between direct and indirect sources of evidence was assessed using global and local approaches. Global inconsistency was assessed using a design-by-treatment test.(8) Local inconsistency was determined by comparing direct and indirect evidence for each pairwise treatment comparison with the node-splitting approach.(4)

No protocol deviations were made after registration in PROSPERO.

**References of Supplemental File 2**

1. Martínez-Vizcaíno V, Amaro-Gahete FJ, Fernández-Rodríguez R, Garrido-Miguel M, Cavero-Redondo I, Pozuelo-Carrascosa DPJSM. Effectiveness of fixed-dose combination therapy (polypill) versus exercise to improve the blood-lipid profile: a network meta-analysis. 2022:1-13.

2. Ware J, Kosinski M, Keller SJAusm. SF-36 physical and mental health summary scales. 1994.

3. Höfer S, Lim L, Guyatt G, Oldridge NJH, outcomes qol. The MacNew Heart Disease health-related quality of life instrument: a summary. 2004;2(1):1-8.

4. Daly C, Dias S, Welton NJ, Anwer S, Ades AE. NICE Guidelines Technical Support Unit. Meta-Analysis. Guideline Methodology Documents. London: National Institute for Health and Care Excellence; 2021.

5. Borenstein M, Hedges LV, Higgins JPT, Rothstein HR. Chapter 4 of the book: Introduction to Meta-Analysis: John Wiley & Sons, Ltd; 2009.

6. Nikolakopoulou A, Mavridis D, Salanti G. Demystifying fixed and random effects meta-analysis. J Evidence-based mental health. 2014;17(2):53.

7. Rücker GJRsm. Network meta‐analysis, electrical networks and graph theory. 2012;3(4):312-24.

8. Higgins JP, Jackson D, Barrett JK, Lu G, Ades AE, White IR. Consistency and inconsistency in network meta-analysis: concepts and models for multi-arm studies. Res Synth Methods. 2012;3(2):98-110.
